# The frequency of differentiated CD3^+^CD27^-^CD28^-^ T cells predicts response to CART cell therapy in diffuse large B-cell lymphoma

**DOI:** 10.3389/fimmu.2022.1004703

**Published:** 2023-01-09

**Authors:** Nina Worel, Katharina Grabmeier-Pfistershammer, Bernhard Kratzer, Martina Schlager, Andreas Tanzmann, Arno Rottal, Ulrike Körmöczi, Edit Porpaczy, Philipp B. Staber, Cathrin Skrabs, Harald Herkner, Venugopal Gudipati, Johannes B. Huppa, Benjamin Salzer, Manfred Lehner, Nora Saxenhuber, Eleonora Friedberg, Philipp Wohlfarth, Georg Hopfinger, Werner Rabitsch, Ingrid Simonitsch-Klupp, Ulrich Jäger, Winfried F. Pickl

**Affiliations:** ^1^ Department of Blood Group Serology and Transfusion Medicine, Medical University of Vienna, Vienna, Austria; ^2^ Institute of Immunology, Center for Pathophysiology, Infectiology and Immunology, Medical University of Vienna, Vienna, Austria; ^3^ Department of Medicine I, Division of Hematology and Hemostaseology, Medical University of Vienna, Vienna, Austria; ^4^ Department of Emergency Medicine, Medical University of Vienna, Vienna, Austria; ^5^ Institute for Hygiene and Applied Immunology, Center for Pathophysiology, Infectiology and Immunology, Medical University of Vienna, Vienna, Austria; ^6^ Christian Doppler Laboratory for Next Generation CAR T Cells, St. Anna Children´s Cancer Research Institute, Vienna, Austria; ^7^ Department of Medicine I, Division of Blood and Bone Marrow Transplantation, Medical University of Vienna, Vienna, Austria; ^8^ Department of Pathology, Medical University of Vienna, Vienna, Austria

**Keywords:** diffuse large B cell lymphoma, chimeric antigen receptor T cells therapy, CD27, CD28, biomarker

## Abstract

**Background:**

Chimeric antigen receptor T (CART) cell therapy targeting the B cell specific differentiation antigen CD19 has shown clinical efficacy in a subset of relapsed/refractory (r/r) diffuse large B cell lymphoma (DLBCL) patients. Despite this heterogeneous response, blood pre-infusion biomarkers predicting responsiveness to CART cell therapy are currently understudied.

**Methods:**

Blood cell and serum markers, along with clinical data of DLBCL patients who were scheduled for CART cell therapy were evaluated to search for biomarkers predicting CART cell responsiveness.

**Findings:**

Compared to healthy controls (n=24), DLBCL patients (n=33) showed significant lymphopenia, due to low CD3^+^CD4^+^ T helper and CD3^-^CD56^+^ NK cell counts, while cytotoxic CD3^+^CD8^+^ T cell counts were similar. Although lymphopenic, DLBCL patients had significantly more activated HLA-DR^+^ (P=0.005) blood T cells and a higher frequency of differentiated CD3^+^CD27^-^CD28^-^ (28.7 ± 19.0% versus 6.6 ± 5.8%; P<0.001) T cells. Twenty-six patients were infused with CART cells (median 81 days after leukapheresis) and were analyzed for the overall response (OR) 3 months later. Univariate and multivariate regression analyses showed that low levels of differentiated CD3^+^CD27^-^CD28^-^ T cells (23.3 ± 19.3% versus 35.1 ± 18.0%) were independently associated with OR. This association was even more pronounced when patients were stratified for complete remission (CR versus non-CR: 13.7 ± 11.7% versus 37.7 ± 17.4%, P=0.001). A cut-off value of ≤ 18% of CD3^+^CD27^-^CD28^-^ T cells predicted CR at 12 months with high accuracy (P<0.001). *In vitro*, CD3^+^CD8^+^CD27^-^CD28^-^ compared to CD3^+^CD8^+^CD27^+^CD28^+^ CART cells displayed similar CD19^+^ target cell-specific cytotoxicity, but were hypoproliferative and produced less cytotoxic cytokines (IFN-γ and TNF-α). CD3^+^CD8^+^ T cells outperformed CD3^+^CD4^+^ T cells 3- to 6-fold in terms of their ability to kill CD19^+^ target cells.

**Interpretation:**

Low frequency of differentiated CD3^+^CD27^-^CD28^-^ T cells at leukapheresis represents a novel pre-infusion blood biomarker predicting a favorable response to CART cell treatment in r/r DLBCL patients.

## Introduction

Diffuse large B cell lymphoma (DLBCL) represents the most frequent form of non-Hodgkin’s lymphoma (NHL). Five-year survival rates range from 55% to 64% (1, 2); however, patients who experience early relapse, or who are refractory to initial immunochemotherapy have a poor prognosis (3). In fact, salvage therapy for patients with refractory NHL has been associated with frequent therapy failures (>70%) and poor long-term outcome with an overall survival of only 6 months (3). Even consolidation therapy with subsequent autologous stem cell transplantation leads to only 50% long-term survival (4, 5).

Chimeric antigen receptor T (CART) cells represent a novel treatment option for patients with refractory/relapsing (r/r) DLBCL (6, 7). CART cell therapy takes advantage of autologous peripheral blood (PB) T cells, which are genetically modified *ex vivo* to express a chimeric antigen receptor (CAR) designed to target the CD19 antigen on the surface of the malignant B cell clone (8). Despite initial promising results with tisagenlecleucel (formerly CTL019) (7, 9), axicabtagene ciloleucel (formerly KTE-C19) (10) and lisocabtagene maraleucel (formerly JCAR017) (11), leading to overall response (OR) and complete remission (CR) rates of 83% to 52% and 58% to 40% (7, 11, 12), respectively, clearly not all patients benefit from CART cell therapy in the long-term (7). In fact, response rates decline to approximately 32% after one year (7). However, the identification of patients most likely to benefit from CART cell therapy is difficult to achieve by solely using clinical and basic laboratory criteria. Therefore, a reliable predictor of response to CART cell therapy at the time of enrollment, e.g., by a simple blood test, is an unmet need for optimal patient selection (13).

Currently, the best predictors of responsiveness to CART cell therapy are low lactate dehydrogenase (LDH) levels after lymphodepletion before CART cell infusion, a low tumor volume and a low Eastern Cooperative Oncology Group (ECOG) performance status (7, 14–16). Owing to the mode of action of CART cells, the immune system most likely plays a major role in its effectiveness. However, all three markers (LDH, tumor volume, ECOG) are not directly related to the immune system, and thus can, at best, represent surrogate markers for future tumor-immune surveillance by the gene modified autologous CART cells. More recently, other factors strongly linked to the immune system and possibly impacting on the response to CART cell therapy have been suggested. These factors include, but are not restricted to: i) defective T cell function (poor initial “pre-CAR” T cell quality or decreasing “post-CAR” T cell function) (17); ii) microenvironmental suppression (check point inhibition and suppressive cytokines) (18); and iii) antigen escape (target antigen modulation (19) or myeloid lineage switch) (20). In addition, increased frequencies of CD27^+^CD45RO^-^CD8^+^ T cells at the time of leukapheresis have been implicated to correlate with sustained remission in patients with chronic lymphocytic leukemia treated with CD19 CART cells (21), in multiple myeloma patients treated with B cell maturation antigen (BCMA)-specific CART cells (22), and recently in patients with DLBCL (23). It has been suggested that CD27^+^CD45RO^-^CD8^+^ T cells belong to the group of antigen-experienced CD3^+^CD8^+^ T lymphocytes that have long-lasting memory capabilities and improved ability to expand *in vitro* and *in vivo* (21, 22, 24). While of interest, the respective marker combination does not define a single cellular phenotype since CD45RO negativity may identify both naïve CD8^+^ T cells as well as antigen-experienced “stem cell memory” cells (23). Moreover, focusing the analyses exclusively on CD8^+^ T cells has the problem of potentially underestimating the cytotoxic potency of CD4^+^ T cells turned into CART cells during the manufacturing process. However, it has been clearly shown in adoptive T cell transfer studies in preclinical melanoma models that more differentiated CD8^+^ effector T cells are less effective for *in vivo* tumor treatment and that the renewal capacity of CD8^+^ T cells as determined by their telomer length plays an important role in that respect (25). In line with these studies, adoptive T cell transfer studies with autologous CD8^+^CD27^+^ T cells led to durable responses in heavily pretreated patients with metastatic melanoma (26). Apart from phenotypic data, a recent study suggested that germline mutations in UNC13D and compound heterozygous forms of CXCR1 may represent additional resistance factors to CART therapy (17). Whether and how they correlate with the cell surface phenotype of CD3^+^ T cells remains to be shown in the future. Furthermore, it should also be noted that for many patients, it is not possible to generate a suitable CART cell product due to prolonged lymphopenia and the associated inability to isolate a sufficient number of functional T cells (27).

Lymphopenia, as well as poor T cell quality and function, may reflect the intensity of previous immuno-chemotherapies, but may also result from hyperactivation of T cells, a process well-known to lead to their subsequent hypoproliferation and reduced life expectancy. Hyperactivated HLA-DR^+^ T cells have been shown to down-modulate cell surface expression of the co-stimulatory molecules CD27 and CD28 (28–31), which are otherwise decisively involved in the regulation of T cell activation (32, 33), the formation and maintenance of antigen-experienced T cells (34) and tumor immune surveillance (35). However, increased frequencies of HLA-DR^+^ T cells may also be the result of homeostatic proliferation (36). The expression levels of CD27 and CD28 as well as those of the high molecular weight form of CD45, i.e., CD45RA, and the chemokine receptor CCR7 allow, in principle, the determination of the position of a given T cell within the linear T cell differentiation model proposed by Romero et al. (37). In that model, CD3^+^CD27^-^CD28^-^ T cells are mainly composed of T effector memory cells re-expressing CD45RA (TEMRA) cells and to a lower degree also contain effector memory type 3 (EM3) cells. While CCR7 is a robust marker for distinguishing between central and effector memory T cells, CD45RA is somewhat problematic because it is expressed on both naïve and terminally differentiated TEMRA cells and is overexpressed in 1 of 20 Caucasian individuals due to the C77G mutation (38), making it much more difficult to distinguish between *bona fide* CD45RA^+^ and CD45RA^-^ cell subsets. Therefore, we here analyzed leukocyte subset distribution, T cell activation, and focused on CD27 and CD28 expression of bulk CD3^+^ T cells in the blood and corresponding leukapheresis products of adult r/r DLBCL patients and correlated the results with 3 months OR to CART cell therapy.

## Patients and methods

### Patients and clinical trial conduct

Between January 2016 and January 2022, 33 patients diagnosed with r/r DLBCL and scheduled for treatment with CART cells at our institution were enrolled into this study to investigate the composition of leukocyte subpopulations, their activation and differentiation status, together with serum markers in peripheral blood (PB) and leukapheresis samples. Patients gave their written informed consent in accordance with the Declaration of Helsinki. Patients received CART cells in clinical trials with tisagenlecleucel (n=15; Ethics Committee (EC) No.: 1422/2015, 1607/2018), YTB323 [n=2; EC No.: 2055/2019 (39)], or in routine applications of tisagenlecleucel (n=6) or axicabtagene ciloleucel (n=3). Analysis of data was approved by the EC of the Medical University of Vienna (EC No.: 1290/2020). Patient characteristics are presented in **Table 1** and **S1**. Of the 33 enrolled patients, 26 already received CART cells, more than 3 months previously, at the time of data cut-off of this study. Seven patients were excluded from the study because they died before CART cell infusion (n=5), or received another treatment (n=2). The patients included into this study were heavily pretreated, showing failure to respond to two or more treatment lines, thus representing the subpopulation of patients with relapsed DLBCL eligible for CART therapy. The healthy control subjects (n=24) were age- (median 60 years; range 33-77 years) and sex- (10 women; 41.7%) matched and similar to the patients of Caucasian ethnicity.

**Table 1:** Demographics, pathological features and clinical performance of patients.

	All DLBCL patients enrolled in study	Patients who received CART cell treatment (n=26; 78.8%)					
		3 mos responders(CR+PR)	3 mos non-responders	P-value	3 mos CR	3 mos non-CR	P-value
No. of DLBCL patients (%)	33 (100)	15 (45.5)	11 (33.3)		11 (33.3)	15 (45.5)	
Demographics, disease type and clinical presentation							
Demographics							
Age in years, median (range)	61.8 (32.9-77.2)	67.5 (36.1-77.2)	48.9 (32.9-66.5)	0.003^*^	65.6 (36.1-77.2)	53.8 (32.9-74.9)	0.07^*^
Gender, female (%)	14 (42.4)	8 (53.3)	4 (36.4)	0.45	5 (45.5)	6 (46.7)	1.00
Disease Form							
Bulky (total)	11 (35.5)	3 (14)	5 (11)	0.39	1 (10)	7 (46.7)	0.09
Pathological features							
Double/triple Hit (total)	21 (27)	9 (10)	9 (11)	1	6 (6)	12(15)	0.53
Molecular biological features							
*MYC rearrangement* positive by FISH (total)	16 (28)	5 (12)	8 (11)	0.21	4 (9)	9 (14)	0.68
*BCL-2 rearrangement* positive by FISH (total)	22 (27)	10 (11)	8 (11)	0.59	8 (8)	10 (14)	0.28
*BCL-6 rearrangement* positive by FISH (total)	16 (22)	6 (8)	8 (10)	1.00	5 (6)	9 (12)	1.00
Cell of origin GCB (total)	15 (30)	5 (13)	8 (11)	0.12	3 (9)	10 (15)	0.21
Double-hit score 2 (accord. *Green et al.)*	19 (30)	6 (13)	10 (11)	0.03	3 (9)	13 (15)	0.02
Clinical performance at relapse							
IPI (international prognostic index), median (range)	2 (0-4)	2 (0-4)	2 (0-2)	0.89**	1.5 (0-2)	2 (0-4)	0.09**
IPI (age adjusted), median (range)	2 (0-3)	1 (0-3)	2 (0-2)	1.00**	1 (0-2)	2 (0-3)	0.07**
Ann-Arbor staging, median (range)	3 (1-4)	3 (1-4)	2 (1-4)	0.42**	2 (1-4)	3 (1-4)	0.46**
ECOG performance status: 0 (1)	25 (8)	12 (3)	8 (2)	1.00	8 (3)	12 (2)	0.62
Pretreatment							
No. prior treatment lines pre leukapheresis, median (range)	3 (1-11)	3 (1-11)	3 (1-6)	0.85	3 (1-11)	3 (1-6)	0.87
<4 treatment lines (total)	25 (23)	13 (15)	8 (11)	0.62	9 (12)	12 (15)	1.00
Laboratory parameters							
At leukapheresis							
LDH (<245 U/L), mean ± SD	325.9 ± 180.3	236.1 ± 114.0	366.1 ± 162.5	0.02^*^	246.4 ± 132.2	323.9 ± 155.9	0.19^*^
CRP (<0.5 mg/L), mean ± SD	2.6 ± 4.2	0.8 ± 0.8	3.2 ± 5.0	0.07^*^	0.7 ± 0.9	2.6 ± 4.3	0.16^*^
Fibrinogen (200-400 mg/dL), mean ± SD	476.7 ± 155.2	424.5 ± 114.5	465.2 ± 11.7	0.39^*^	405.4 ± 99.6	467.1 ± 120.3	0.19^*^
B2M (0.8-2.2 mg/L), mean ± SD	2.8 ± 0.9	2.8 ± 1.1	2.8 ± 0.7	0.85^*^	2.8 ± 1.2	2.8 ± 0.7	0.96^*^
At CART infusion							
LDH (<245 U/L), mean ± SD	354.7 ± 394.6	234.9 ± 92.9	518.2 ± 560.0	0.06^*^	232.3 ± 103.4	444.5 ± 490.9	0.17^*^
B2M (0.8-2.2 mg/L), mean ± SD	3.0 ± 1.0	2.6 ± 0.8	3.6 ± 1.1	0.02^*^	2.4 ± 0.6	3.5 ± 1.1	0.01^*^

Table shows demographics, pathological features and clinical performance of all patients, and patients stratified according to response at 3 months (CR plus PR) versus non-response. Hypothesis testing using normally distributed data has been performed with the Student’s t-test, while for categorized data such as demographic data like sex, cytogenetic marker positivity, the Fisher’s Exact test was used. Non-normally ordinally distributed data were tested by the Mann-Whitney U test. *) P-values calculated with Student’s t-test **) Mann-Whitney U-Test, all other P-values calculated with Fisher’s exact test; CR, complete remission; PR, partial remission; Hb, Hemoglobin; Plt, platelets; LDH, lactate dehydrogenase; CRP, C-reactive protein; B2M, beta-2-microglobulin; GCB, germinal center B cell; ECOG, Eastern cooperative oncology group score; IPI, international performance index; mos, months.

### Flow cytometric analyzes

Immunophenotyping of PB and the leukapheresis products was performed with fresh samples according to standard procedures (40) using directly conjugated monoclonal antibodies (**Supplemental Table S2** **).** To keep numbers of flow cytometric parameters low, the CD27 and CD28 expression status was analyzed on bulk CD45^+^CD3^+^ T cells. Acquisition was performed on flow cytometers (FACS Calibur and LSR Fortessa, Becton Dickinson, San Jose, CA; Navios or Cytoflex, Beckman Coulter, Krefeld, Germany) supported by the Cellquest, Diva and Kaluza software, respectively. Acquired data were analyzed with Flow Jo software (BD).

### Generation of CART cells for *in vitro* studies

Buffy coats from anonymous healthy donor’s blood were purchased from the Austrian Red Cross, Vienna. CD3^+^ primary human T cells were isolated using the RosetteSep Human T cell Enrichment Cocktail (STEMCELL Technologies, Vancouver Canada) and immediately cryopreserved in RPMI-1640 GlutaMAX medium (Thermo Fisher Scientific, Waltham, MA) supplemented with 20% FCS and 10% DMSO (both from Merck, Darmstadt, Germany). Primary human T cells were thawed in RPMI-1640 GlutaMAX medium, supplemented with 10% FCS, 1% penicillin-streptomycin (Thermo Fisher Scientific) and 200 IU mL^-1^ recombinant human IL-2 (Peprotech, Waltham, MA) and activated with Dynabeads Human T-Activator CD3/CD28 beads (Thermo Fisher Scientific) at a 1:1 ratio according to the manufacturer’s instructions. Twenty-four hours after stimulation, T cells were transduced in cell culture plates, which were coated with RetroNectin (Takara, Shiga, Japan), according to the manufacturer’s instructions. Thawed lentiviral supernatant was added to the T cells at a final dilution of 1:2, yielding a cell concentration of 0.5 x 10^6^ cells mL^-1^. Forty-eight hours after transduction, selection of CART cells was initiated by treatment with 1 µg mL^-1^ puromycin (Merck, Germany) for two days. Transduced T cells were cultivated in AIM V medium (Thermo Fisher Scientific) supplemented with 2% Octaplas (Octapharma, Vienna, Austria), 1% L-glutamine, 2.5% HEPES (both from Thermo Fisher Scientific) and 200 IU mL^-1^ recombinant human IL-2 for 14 days and then frozen in liquid nitrogen in IMDM medium containing 20% FB and 10% DMSO until further use.

### Construction of lentiviral vector

VSV-G pseudotyped lentivirus was generated by co-transfection of Lenti-X 293T cells (Takara) with a puromycin-selectable pCDH expression vector (System Biosciences, USA) encoding the second-generation anti-CD19-CAR (FMC63.4-1BB.ζ) and viral packaging plasmids pMD2.G and psPAX2 (Addgene plasmids #12259 and #12260, respectively; kind gifts from Didier Trono) using the PureFection Transfection Reagent (System Biosciences, Palo Alto, CA) according to the manufacturer’s instructions. Viral supernatants were collected on day 2 and 3 after transfection and were concentrated 100-fold using the Lenti-X Concentrator (Takara) according to the manufacturer’s instructions. Viral suspensions were frozen at -80°C until further use.

### Functional *in vitro* assays with CART cells

For *in vitro* experiments, CART cells were gently thawed and cultured in AIMV medium (Thermo Fisher Scientific, USA) supplemented with 2% Octaplas (Octapharma), 1% L-glutamine, 2.5% HEPES (both from Thermo Fisher Scientific, USA) and 50 IU mL-1 recombinant human IL-2 (Peprotech). One day after thawing, CART cells were expanded by adding five times the number of irradiated (120 Gray) TM-LCL cells, a human B lymphocyte cell line immortalized by Epstein-Barr virus infection (41), which have been optimized as feeder cells for CD19 CART cell expansion (42). Expansion of CD19 CART cells after removal of CD3CD28-beads with CD19^+^LCL cells has been used in the past and represents an accepted procedure for CART cell expansion and propagation (43). After three days, cells were further expanded every two to three days by adding fresh medium in a 1:2 ratio. Ten days after expansion, CART cells were FACS sorted with the antibodies listed in **Table S2** to obtain CD3^+^CD8^+^CD27^+^CD28^+^, CD3^+^CD8^+^CD27^-^CD28^-^ T, CD3^+^CD4^+^CD27^+^CD28^+^ and CD3^+^CD4^+^CD27-CD28^-^ cell populations on a Sony SH800 Sorter (Sony Biotechnology, San Jose, CA) and cultured in the presence of IL-2 in medium as described above. Five to seven days later, cells were used for *in vitro* assays. For proliferation assays, 1 x 10^5^ CART cells were incubated with the indicated amounts of irradiated (120 Gray) CD19^+^ TM-LCL cells (ranging from 2 x 10^5^ to 1 x 10^4^ cells) in triplicates in 96-well round-bottom tissue culture plates (Sarstedt, Nümbrecht, Germany) in a total volume of 200 µl for 48 h. Cells were pulsed with [methyl-^3^H]-thymidine (1 µCi per well) for 18 hours and thymidine up-take was analyzed as previously described (44). For analysis of T cell activation and cytokine production, 1 x 10^5^ CART cells were incubated with the indicated amounts of CD19^+^ TM-LCL cells (ranging from 2 x 10^5^ to 1 x 10^4^ cells) in triplicates in 96-well round-bottom plates in a total volume of 200 µl for 72 hours. Subsequently, cell suspensions were transferred to 1.5 ml microcentrifuge tubes, centrifuged at 600 g for 5 minutes, supernatants were collected and subjected to cytokine analyses with a cytometric bead array (Luminex, Austin, TX) as described previously (45). Cells were stained as described (44), acquired on a Cytoflex flow cytometer (Beckmann Coulter) and data analyzed with the Flow Jo software package (Becton Dickinson).

For cytotoxicity assays, 1 x 10^6^ CD19^+^ TM-LCL or CD19^-^ K562 cells were resuspended in 50 µl of culture medium each and labelled with 50 µl of Na^51^CrO_4_ (Perkin Elmer, Boston, MA) at 37°C for 1 hour. After four subsequent washes, 5 x 10^3^ TM-LCL and K562 cells were seeded into individual wells of 96-well round-bottom tissue-culture plates and incubated with the indicated amounts of sorted CART cells in duplicates/triplicates. Medium or 2% triton-X100 was added to target cells to determine spontaneous and maximum release, respectively. Subsequently, plates were centrifuged at 100 g for 5 minutes and incubated at 37°C for 5 hours. Supernatants were then collected with the Skatron system (Molecular Devices, Biberach an der Riss, Germany) and radioactivity was determined on a Cobra II gamma-counter (Packard, Meriden, CT). The percentage of specific release was determined as follows [CART cell induced release (cpm) – spontaneous release (cpm)]/[maximum release (cpm) - spontaneous release (cpm)]x100.

### Statistics

The study was designed as a cohort study. Response to CART cell treatment was defined as complete response (CR) or partial remission (PR) at three months after CART cell infusion. No response was defined as stable disease (SD) or progressive disease (PD) after receiving CART cells. We present categorized data as absolute counts and relative frequencies, continuous data as mean and standard deviation, or median and range. Where applicable, we log-transformed variables to yield approximate normal distributions. To test the H_0_ of no association of T cell subsets with the outcome to CART cell therapy, the Fisher’s exact test and the independent sample t-test was used. To quantify the association between the outcome overall response at 3 months and the percentage of CD3^+^CD27^-^CD28^-^ T cells, we used exact logistic regression, owing to the limited sample size. We also assessed other predefined variables and added these variables as co-variables into the main model separately. Generally, a two-sided P-value <0.05 was considered statistically significant.

### Data Sharing

Please contact Dr. Nina Worel for sharing of data at nina.worel@meduniwien.ac.at.

## Results

### Enrollment and clinical characterization of r/r DLBCL patients

Our study aimed to identify robust pre-infusion biomarkers in the blood and leukapheresis samples of r/r DLBCL patients, as possible predictors to the subsequent response to CART cell therapy. Accordingly, between January 2016 and January 2022, 33 patients with r/r DLBCL were enrolled into this cohort study (**Figure S1**). Patients consisted of 19 males and 14 females, with a median age of 61.8 years (range, 32.9 to 77.2 years, **Table 1**) and a median disease duration at leukapheresis of 18.0 months (range, 3.7-266.4 months) (**Table S1**). Median time from PB assessment at the time of leukapheresis to CART cell infusion was 3.3 months (range, 1.2 to 14.1 months). These patients had a median follow-up time of 15.5 months (range, 6.1 to 57.1 months). OR at 3 months was observed in 15 patients (57.7%), with 11 patients achieving a CR (42.3%).

### Cellular parameters of r/r DLBCL patients at leukapheresis

**Figure 1:**
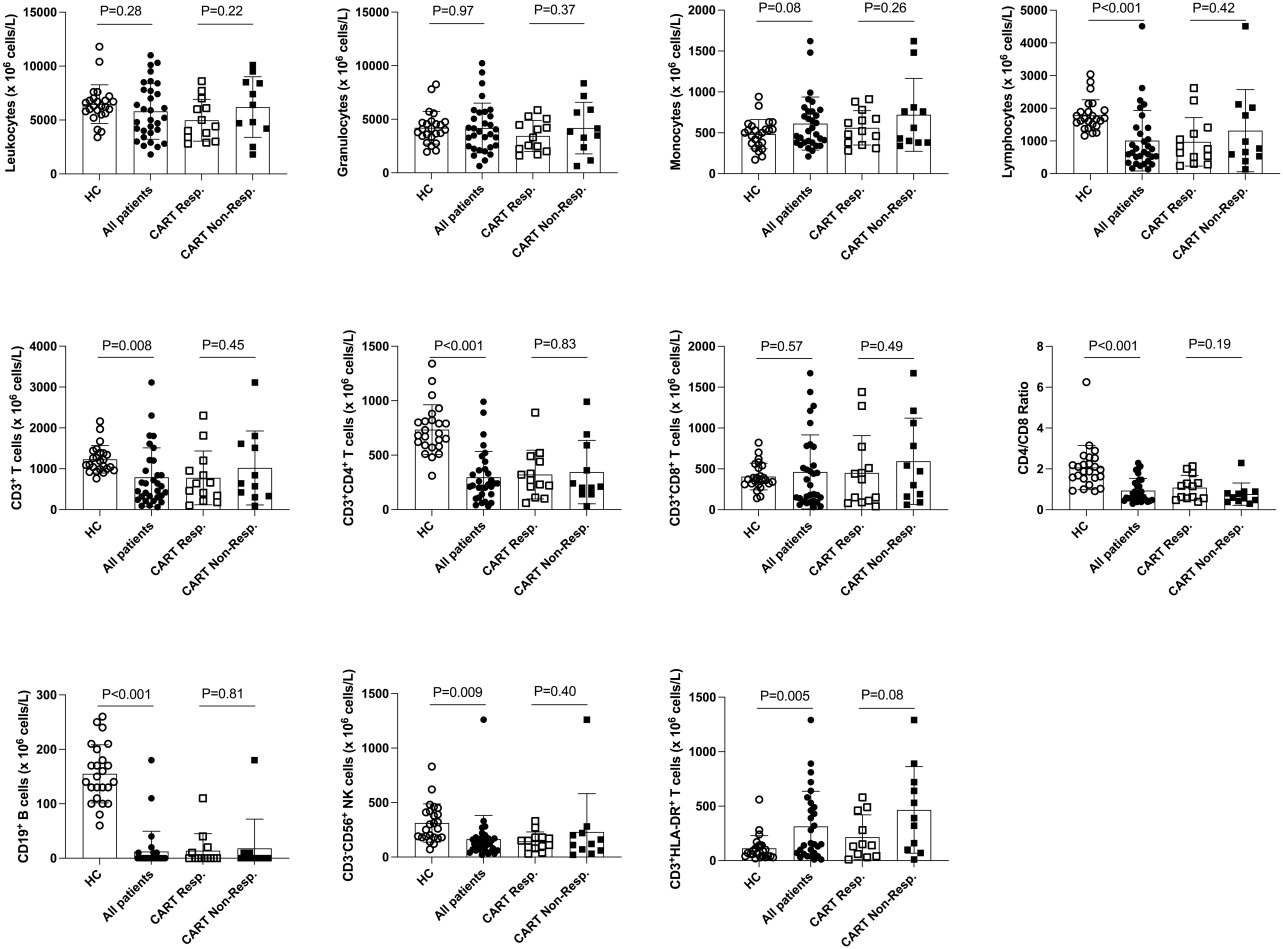
Distribution of leukocyte populations in the PB of healthy control subjects, total r/r DLBCL patients and CART cell recipients. Here, the distribution of PB cell populations of 24 healthy control subjects (HC) and 31 of 33* r/r DLBCL patients is shown. Data of 13 of 15* CART cell responders (except 11 for CD3^+^HLA-DR^+^ T cells) and 11 CART non-responders 3 months after CART therapy are shown separately. P-values (unpaired t-test) are indicated. *) PB of two patients belonging to the CART responders was not available for analyses at this stage.

**Figure 2:**
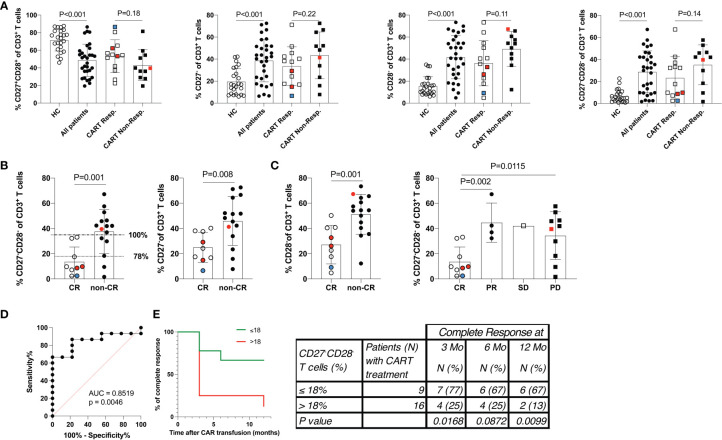
Low frequency of differentiated CD3^+^CD27^-^CD28^-^ T cells predicts a favorable response to CART cell therapy. **(A)** The distribution of PB CD3^+^ T cell populations stratified by the CD27 and CD28 expression status is given. Data show 24 healthy control subjects (HC), 31 of 33* r/r DLBCL patients scheduled for CART cell therapy, and more detailed data for 13 of 15* CART cell responders and all non-responders (n=11). **(B)** Shows the distribution of PB CD3^+^ T cell populations stratified by the CD27 and CD28 expression status in patients who were further separated into 9 of 11* complete responders and compared to 15 non-complete responders. Horizontal lines at 18% of CD3^+^CD27^-^CD28^-^ T cells indicate the 78% (dotted line) and at 35% of CD3^+^CD27^-^CD28^-^ T cells indicate 100% (dashed-and-dotted line) specificity levels (at sensitivity levels of 87% and 67%, respectively) of numbers of CD3^+^CD27^-^CD28^-^ T cell numbers to predict CR **(C)** Shown are the numbers of CD3^+^CD27^-^CD28^-^ T cells of patients who achieved complete remission (CR), partial remission (PR), stable disease (SD) or progressive disease (PD). P-values (unpaired Student’s t-test) are indicated. Cell frequencies were determined in 31 of 33* patients. Patients were treated with tisagenlecleucel (white/black symbols), axicabtagene ciloleucel (red symbols) or YTB323 (blue symbols). *) PB of two patients belonging to the CART responders was not available for analyses at this stage. **(D)** ROC (receiver operator characteristics) curve indicating the performance of numbers CD3^+^CD27^-^CD28^-^ T cells for classifying CR. **(E)** Duration of complete remission (CR) after CART cell therapy. Shown are the percent of patients presenting with CR over the observational period of 12 months (Mo) with staging at 0, 3, 6 and 12 months. Patients were stratified according to those with >18% or ≤18% of CD3^+^CD27^-^CD28^-^ T cells at the time of leukapheresis. Table shows the number (N) of patients within each group at each time point (percent of group in parenthesis). P values were calculated with Fisher’s exact test.

First, we assessed PB leukocyte subpopulations at the time of leukapheresis (**Table 2** and **Figure 1**). Remarkably, r/r DLBCL patients had significant lymphopenia compared to healthy controls (HC) (1009 ± 927 x10^6^/L versus 1785 ± 478 x10^6^/L; P<0.001), due to reduced CD3^+^CD4^+^ T helper (297 ± 236 x10^6^/L versus 735 ± 229 x10^6^/L; P<0.001) and CD3^-^CD56^+^ NK cell numbers (164 ± 218 x10^6^/L versus 313 ± 176 x10^6^/L; P=0.009). CD3^+^CD8^+^ T cell, NKT cell, neutrophil and overall leukocyte numbers were similar to HC (**Table 2** and **Figure S2**). The CD3^+^CD4^+^ lymphopenia led to a significantly lower CD4/CD8-ratio (0.9 ± 0.6 versus 2.1 ± 1.1, P<0.001) in DLBCL patients. Moreover, patients’ T cells had clear signs of activation, as determined by HLA-DR co-expression (315 ± 322 x10^6^/L versus 113 ± 116 x10^6^/L; P=0.005). Notably, chronic activation of T cells may lead to cell differentiation and replicative senescence, which is frequently accompanied by downregulation of the co-stimulatory molecules CD27 and CD28 (30, 31), the acquisition of memory (CD45RO/RA) and the loss of lymphnode homing (CCR7) markers (37). Indeed, when we examined the overall study population of r/r DLBCL patients in that regard, we found significantly higher percentages of differentiated CD3^+^CD27^-^CD28^-^ (28.7 ± 19.0% versus 6.6 ± 5.8%; P<0.001), CD3^+^CD27^-^ (38.6 ± 19.2% versus 19.6 ± 11.9%; P<0.001) and CD3^+^CD28^-^ (41.7 ± 19.6% versus 15.5 ± 8.5%; P<0.001) PB T cells when compared to age-matched HC (**Table 3**; **Figures 2A** and **S3**). CD3^+^CD27^-^CD28^-^ consisted exclusively of highly differentiated CCR7^-^ CD45RA^-/+^ T effector memory (EM)/T effector memory RA cells (TEMRA) (**Figure S4**). Not unexpectedly, almost complete B cell aplasia was seen in most DLBCL patients (P<0.001).

**Table 2:** Differences in lymphocyte populations between r/r DLBCL patients and healthy controls.

				CART cell recipients analyzed (n=24; 72.7%)		
N (%)	DLBCL patients enrolled in study^§^ 31 (94.0)	Healthy controls 24 (100.0)	P-value^$^	3 mos CART responders (CR+PR) 13 (39.4)^§^	3 mos CART non-responders 11 (33.3)	P-value^$^
Leukocytes	5803 ± 2579*	6475 ± 1792*	0.28	4977 ± 1938*	6209 ± 2810*	0.22
Neutrophils	4183 ± 2321	4207 ± 1530	0.97	3445 ± 1454	4177 ± 2406	0.37
Monocytes	613 ± 324	483 ± 179	0.08	561 ± 211	720 ± 446	0.26
Lymphocytes	1009 ± 927	1785 ± 478	<0.001	972 ± 743	1315 ± 1258	0.42
CD3^+^ T cells	791 ± 722	1232 ± 337	0.008	777 ± 658	1020 ± 904	0.45
CD3^+^CD4^+^ T cells	297 ± 236	735 ± 229	<0.001	322 ± 223	345 ± 292	0.83
CD3^+^CD8^+^ T cells	462 ± 453	406 ± 163	0.57	452 ± 456	594 ± 528	0.49
CD4/CD8-Ratio	0.9 ± 0.6	2.1 ± 1.1	<0.001	1.1 ± 0.6	0.8 ± 0.5	0.19
CD19^+^ B cells	12 ± 37	155 ± 53	<0.001	14 ± 31	18 ± 54	0.81
CD3^-^CD56^+^ NK cells	164 ± 218	313 ± 176	0.009	144 ± 86	230 ± 351	0.40
CD3^+^HLA-DR^+^ T cells	315 ± 322	113 ± 116	0.005	215 ± 205	465 ± 397	0.08
CD3^+^CD56^+^ NKT cells (%)	12.4 ± 9.4	11.2 ± 9.1	0.65	11.8 ± 10.6	14.0 ± 9.8	0.63

Shown are leukocyte and lymphocyte counts of DLBCL patients enrolled into the study compared to age and sex matched healthy control individuals. Patients were stratified according to response at 3 months (CR plus PR) versus patients with no-response. ^$)^ Statistical differences between collectives were determined by Student’s t-test. ^§)^ The peripheral blood of two patients belonging to the CR group could not be analyzed. ^*)^ Data show absolute counts x10^6^ cells/L as mean ± standard deviation of the respective populations, except for CD3^+^CD56^+^ NKT cells, for which relative numbers of lymphocytes are given; CART, chimeric antigen receptor T cells; CR, complete remission; PR, partial remission; mos, months.

**Table 3:** PB and leukapheresis material of r/r DLBCL patients scheduled for CART cell therapy contain significantly more CD3^+^ T cells with a differentiated CD27^-^CD28^-^ phenotype when compared to healthy control individuals.

	CD3^+^CD27^+^CD28^+^	CD3^+^CD27^-^	CD3^+^CD28^-^	CD3^+^CD27^+^CD28^-^	CD3^+^CD27^-^CD28^+^	CD3^+^CD27^-^CD28^-^
Peripheral blood						
All r/r DLBCL patients PB (n=31; 94.0%)^§^	48.7 ± 18.4^*^	38.6 ± 19.2	41.7 ± 19.6	13.0 ± 8.5	9.9 ± 7.3	28.7 ± 19.0
Healthy controls (n=24; 100%)	71.5 ± 12.3	19.6 ± 11.9	15.5 ± 8.5	8.9 ± 5.1	13.0 ± 7.8	6.6 ± 5.8
P-value^$^	<0.001	<0.001	<0.001	0.042	0.05	<0.001
CART responders (CR+PR) at 3 months (n=13; 39.4%) ^§^	53.4 ± 18.6	33.5 ± 17.7	36.4 ± 20.6	13.1 ± 10.7	10.3 ± 6.0	23.3 ± 19.3
CART non-responders at 3 months (n=11; 33.3%)	43.2 ± 17.4	43.5 ± 21.2	49.2 ± 15.9	14.1 ± 7.7	8.4 ± 5.5	35.1 ± 18.1
P-values	0.18	0.22	0.11	0.79	0.43	0.14
CR at 3 months (n=9; 27.3%) ^§^	61.5 ± 14.3	25.1 ± 12.0	27.2 ± 15.3	13.5 ± 12.0	11.3 ± 6.8	13.7 ± 11.7
Non-CR at 3 months (n=15; 45.5%)	41.0 ± 16.6	45.9 ± 19.4	51.3 ± 15.8	13.6 ± 7.7	8.3 ± 4.8	37.7 ± 17.4
P-values	0.006	0.008	0.001	0.97	0.21	0.001
Leukapheresis material						
CART Responders (CR+PR) at 3 months (n=15; 45.5%)	54.8 ± 15.4	32.3 ± 15.1	34.6 ± 17.4	12.9 ± 10.4	10.6 ± 6.1	21.7 ± 16.6
CART non-responders at 3 months (n=11; 33.3%)	42.7 ± 19.3	43.5 ± 21.6	48.3 ± 17.8	13.8 ± 7.4	9.1 ± 5.7	34.5 ± 18.8
P-values	0.09	0.13	0.06	0.80	0.51	0.08
CR at 3 months (n=11; 33.3%)	60.1 ± 13.5	26.3 ± 11.9	28.7 ± 15.2	13.5 ± 11.5	11.2 ± 6.9	15.2 ± 12.6
Non-CR at 3 months (n=15; 45.5%)	42.0 ± 17.1	44.9 ± 19.1	49.0 ± 16.2	13.1 ± 7.2	9.1 ± 5.1	35.8 ± 17.2
P-values	0.008	0.009	0.004	0.91	0.38	0.003

Shown are relative numbers of CD3^+^ T cells subsets in PB and the leukapheresis material differentially expressing CD27 and CD28. All enrolled and analyzed patients are compared to healthy control individuals. Alternatively patients have been stratified into CART responders (CR plus PR) versus non-responders. Another comparison examines patients with CR versus patients with non-CR. ^§)^ The PB of two patients belonging to the CR group could not be analyzed. ^*)^ data show relative amounts of CD3^+^ T cells; ^$)^ P-values were calculated with Student’s t-test. CART, chimeric antigen receptor T cells; CR, complete remission; PB, peripheral blood, PR, partial remission; PD, progressive disease; SD, stable disease.

### Low frequency of differentiated CD3^+^CD27^-^CD28^-^ PB T cells in r/r DLBCL patients at leukapheresis correlates with OR

Stratification of patients into CART cell responders at 3 months after CART infusion (CR and PR) versus non-responders (SD and PD) revealed that the T cells of the latter group were in particular more activated, as indicated by HLA-DR co-expression (215 ± 205 x10^6^/L versus 465 ± 397 x10^6^/L; P<0.08) (**Table 2**). Accordingly, a higher frequency of differentiated CD3^+^CD27^-^CD28^-^ PB T cells was also associated with non-responsiveness, while a lower frequency of differentiated CD3^+^CD27^-^CD28^-^ PB T cells was a salient feature of patients with OR (35.1 ± 18.1% versus 23.3 ± 19.3%; P=0.14) (**Table 3** and **Figure 2**). This was due to a trend towards lower frequencies of CD3^+^CD27^-^ PB T cells (33.5 ± 17.7% versus 43.5 ± 21.2%; P=0.22) and CD3^+^CD28^-^ PB T cells (36.4 ± 20.6 versus 49.2 ± 15.9; P=0.11) (**Table 3** and **Figure 2A**). We found a tendency of low numbers of CD3^+^CD27^-^CD28^-^ T cells being associated with month 3 OR (odds-ratio 0.97; 95% confidence interval 0.92-1.01; P=0.14; **Figure S5A**). This association remained virtually unchanged after pairwise adjustment for clinical (international prognostic index, double/triple hit mutation, cell of origin, gender, age at leukapheresis, NOS mutations) and PB parameters (LDH levels at CART cell infusion, frequency of CD3^+^CD27^-^CD28^-^ T cells).

### Low frequency of differentiated CD3^+^CD27^-^CD28^-^ PB T cells at leukapheresis identifies patients with a high likelihood for CR

Next, we compared the CD27 and CD28 expression status on PB T cells of 9 of 11 CR patients to 15 patients presenting with non-CR (PR, SD and PD). From two CR patients no PB was available. Notably, a low frequency of CD3^+^CD27^-^CD28^-^ T cells at the time of leukapheresis (13.7 ± 11.7% versus 37.7 ± 17.4%) was significantly associated with CR at month 3 (P=0.001) (**Figure 2B** and **Table 3**). Inclusion of CD3^+^CD27^-^CD28^-^ values of the two patients with missing PB data but available values of the leukapheresis products (i.e., 16.5% and 37.4% of CD3^+^CD27^-^CD28^-^, respectively) changed the strength of the statistical comparison between CR and non-CR only very slightly (p-values 0.002 versus 0.001, respectively). For ease of comparison, the type of CAR used is given in **Figure 2**. Patients with low or high numbers of CD3^+^CD27^-^CD28^-^ T cells were equally distributed in the subgroups treated with different CAR products suggesting that the type of CAR used did not appear to affect CR rates.

Similar to the above analyses obtained with CART cell responders versus non-responders, pairwise adjustment for clinical and PB parameters did not significantly change this association (**Figure S5B**). Both CD3^+^CD27^-^ (25.1 ± 12.0% versus 45.9 ± 19.4%; P=0.008) and CD3^+^CD28^-^ T cells (27.2 ± 15.3 versus 51.3 ± 15.8; P=0.001) contributed to this association (**Table 3**; **Figures 2C** and **S3**). Of note, the residual CD27 expression on the CD27^+^ T cells within the CD3^+^CD28^-^ subset was found to be reduced compared to the one within the CD3^+^CD27^+^CD28^+^ subset. This indicated that the CD3^+^CD28^-^ subgroup had already begun to downregulate also CD27 expression (data not shown). Therefore, determining the double-negative CD27^-^CD28^-^ status of CD3^+^ T cells appeared to be the most robust strategy for enumerating differentiated T cells and also resulted in a moderately better statistical discrimination between CR and non-CR groups (P=0.001 versus P=0.006) when compared to the CD3^+^CD27^+^CD28^+^ subset. To exclude a sampling bias due to the lack of PB samples from the two CR patients, in addition we compared the leukapheresis products of the CR patients with those of the non-CR patients, for whom the full dataset of 11 and 15 patients was available, in terms of their CD3^+^CD27^-^CD28^-^ T cell counts (**Table 3**). Very similar to PB, we found that low frequencies of CD3^+^CD27^-^CD28^-^ T cells (15.2 ± 12.6% versus 35.8 ± 17.2%) were significantly associated with CR at month 3 (P=0.003) also in the leukapheresis product. Receiver operator characteristics (ROC) curve was used to determine the cut-off above which non-CR could be expected. Numbers of CD3^+^CD27^-^CD28^-^ T cells greater 18% or 35% predicted non-CR with 78% or 100% specificity, (**Figure 2D**). Moreover, the cut-off value of ≤ 18% CD3^+^CD27^-^CD28^-^ T cells predicted the duration of response over the subsequent 12-month follow-up period with high accuracy (p<0.001) (**Figure 2E**).

### CD3^+^CD8^+^CD27^-^CD28^-^ are inferior to CD3^+^CD8^+^CD27^+^CD28^+^ CART cells in terms of proliferation and cytotoxic cytokine production, but not regarding target-cell cytotoxicity

**Figure 3:**
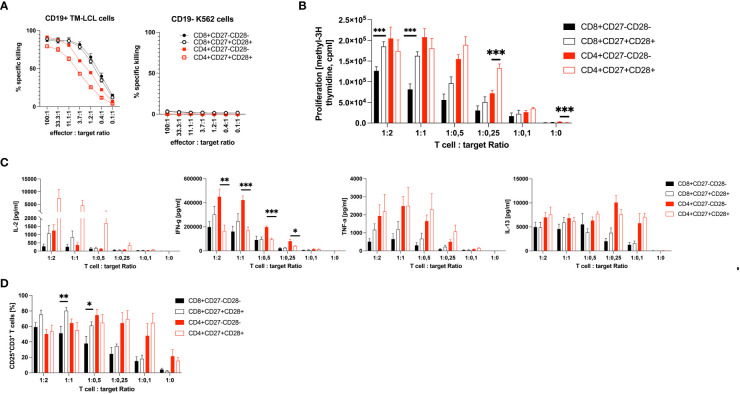
Functional comparison of CD3^+^CD8^+^ and CD3^+^CD4+ CD27^+^CD28^+^ to CD3^+^CD8^+^ and CD3^+^CD4^+^ CD27^-^CD28^-^ CD19 CART cells *in vitro*. Shown is **(A)** the cytotoxic potential as percent specific killing in 5-hour ^51^Cr-release assays, **(B)** the proliferation as count per minutes (cpm) **(C)** the cytokine production in pg/ml and **(D)** the percent expression of the high-affinity IL-2R (CD25) on CD27^-^CD28^-^ CD3^+^CD8^+^ and CD3^+^CD4^+^ T cells in comparison to CD27^+^CD28^+^ CD3^+^CD8^+^ and CD3^+^CD4^+^ T cells upon co-culture with CD19^+^ TM-LCL cells. The cytotoxic potential of CD27^-^CD28^-^ and CD27^+^CD28^+^ T cells in **(A)** is also shown against CD19^-^ K562 cells. X-axes show the effector to target ratios with either a constant amount of 5 x 10^3^ target cells **(A)**, or a constant amount of effector cells of 1 x 10^5^ **(B–D)**. Data are shown as means plus SEM (whiskers). Numbers of CD19 CAR positive T cells were 91.5 ± 0.1% and 96.0 ± 0.3%, respectively. Data show the summary of three **(A**, **D)** or four **(B**, **C)** independent experiments with three different donors in biological triplicates for CD8^+^ T cells, except one donor in duplicates for Cytotoxicity tests, and two independent experiments with two different donors in biological triplicates for CD4^+^ T cells. *P < 0.05, **P < 0.01, ***P < 0.001 as determined by unpaired Student’s t-test.

CD19 CART cells kill malignant and normal CD19^+^ B cells without MHC restriction. CD3^+^CD8^+^ CD19 CART cells have been reported to be able to perform serial killings with higher efficiency and speed than CD3^+^CD4^+^ CD19 CART cells (46). Our above finding that patients with lower numbers of CD3^+^CD27^-^CD28^-^ T cells at leukapheresis have a much better chance of achieving CR, when undergoing CD19-directed CART cell therapy, prompted us to test whether CD3^+^CD8^+^CD27^+^CD28^+^ are, in fact, functionally superior to CD3^+^CD8^+^CD27^-^CD28^-^ CD19 CART cells. Accordingly, we analyzed their cytotoxic, proliferative and cytokine-producing capabilities. Remarkably, CD3^+^CD8^+^CD27^+^CD28^+^ and CD3^+^CD8^+^CD27^-^CD28^-^ CD19 CART cells (expressing the CD19-specific CART cell receptor on 91.6 ± 0.1% % and 91.4 ± 0.1% of CD8^+^ T cells, respectively, **Figure S7**) killed CD19^+^ B cells (TM-LCL) with nearly identical efficacies over the entire range of effector to target (E:T) ratios tested, while no such killing of CD19^-^ K562 cells was observed with either of the two CD3^+^CD8^+^ CD19 CART cell subsets (**Figure 3A**). Notably, also CD3^+^CD4^+^ CART cells (expressing the CD19-specific CART cell receptor on 95.3 ± 0.8% of CD27^-^CD28^-^ and 94.9 ± 2.2% of CD27^+^CD28^+^ CD4^+^ T cells, respectively, **Figure S8**) killed the CD19^+^ B cells (TM-LCL), however, with at least 3- to 6-fold lower efficacy when compared to their CD3^+^CD8^+^ counterparts (**Figure 3A**). Notably, CD4^+^CD27^-^CD28^-^ outperformed CD4^+^CD27^+^CD28^+^ T cells in the killing of CD19^+^ target cells by a factor of 2. However, both CD3^+^CD8^+^CD27^+^CD28^+^ and CD3^+^CD4^+^CD27^+^CD28^+^ CART cells proliferated significantly more efficiently than CD3^+^CD8^+^CD27^-^CD28^-^ and CD3^+^CD4^+^CD27^-^CD28^-^ CART cells, respectively, when co-incubated with CD19^+^ TM-LCL cells at all E:T-ratios tested, with differences ranging between 1.5 ± 0.4 and 2.7 ± 1.7-fold for CD3^+^CD8^+^ and 1.0 ± 0.1 and 1.9 ± 1.1-fold for CD3^+^CD4^+^ T cells (**Figure 3B**). Moreover, CD3^+^CD8^+^CD27^+^CD28^+^ CART cells secreted higher levels of the Th1 cytokines IL-2, IFN-γ and TNF-α, while CD3^+^CD8^+^CD27^-^CD28^-^ CART cells seemed to overproduce the Th2 cytokine IL-13 (**Figure 3C**). The situation was similar for CD3^+^CD4^+^ CART cells, with the sole exception that CD3^+^CD4^+^CD27^-^CD28^-^ as compared to CD3^+^CD4^+^CD27^+^CD28^+^ CART cells produced higher levels of IFN-γ. Notably, the elevated IL-2 secretion levels of CD3^+^CD8^+^CD27^+^CD28^+^ CART cells were paralleled by their increased high-affinity IL-2R (CD25) expression when compared to CD3^+^CD8^+^CD27^-^CD28^-^ CART cells (**Figure 3D**). The limited functional capabilities (i.e., proliferation, IL-2 and TNF-α production both subsets; IFN-γ production for CD8^+^ T cells) of CD27^-^CD28^-^ T cells can be explained by their belonging to the TEMRA and EM3 subsets of memory cells (CCR7^-^CD45RA^+/-^), which are known to have limited renewal capacity (**Figure S4**) (37).

## Discussion

In this cohort study, we aimed to identify a simple and robust pre-infusion blood biomarker to predict the future response to CART cell treatment in r/r DLBCL patients. Compared to HC, r/r DLBCL patients presented with significantly more activated HLA-DR-expressing PB T cells, indicating cellular activation and/or homeostatic proliferation (36), as well as pathologically increased, frequencies of CD3^+^CD27^-^CD28^-^ T cells. According to the linear T cell differentiation model proposed by *Romero et al.* and substantiated by our own analyses (**Figure S4**), T cells with this phenotype belong to the CCR7^-^CD45RA^+/-^ terminally differentiated T effector memory RA (TEMRA) and effector memory type 3 (EM3) cells, respectively (37). We stratified patients according to OR (CR and PR) versus non-response (SD and PD), or CR versus non-CR (PR, SD and PD) 3 months after CART cell treatment, respectively. This revealed that the pathologically high levels of CD3^+^CD27^-^CD28^-^ T cells were associated with non-CR (37.7 ± 17.4%), while patients with CR presented with low, almost physiological, levels of CD3^+^CD27^-^CD28^-^ T cells compared to HC (13.7 ± 11.7% versus 6.6 ± 5.8%). A numeric predictor of CR was determined by plotting a ROC curve, which showed that a cut-off value of ≤18% CD3^+^CD27^-^CD28^-^ T cells (**Figure 2B**) predicted CR with high accuracy even 12 months after CART cell transfusion (**Figure 2E**). This is the first study identifying low numbers of CD3^+^CD27^-^CD28^-^ T cells as a valuable pre-infusion blood biomarker for long-term response to CART cell treatment in r/r DLBCL. Our clinical data corroborate previous *in vitro* findings indicating that both CD27 and CD28 are functionally important co-stimulatory molecules on T cells, which are critically involved in cellular activation programs (32, 47). Moreover, we have demonstrated herein that CD3^+^CD8^+^CD27^-^CD28^-^ CART cells have comparable CD19^+^ target cell killing activity when compared to CD3^+^CD8^+^CD27^+^CD28^+^ CART cells, however, they are clearly inferior regarding CD19^+^ target cell-dependent proliferation and cytotoxic cytokine production, such as IFN-γ and TNF-α (48, 49). Interferon-γ is well-known to contribute to the CART cells’ cytotoxicity by targeting and destroying the tumor stroma (48), while TNF-α has been shown to sensitize tumor cells themselves for getting killed by CD8^+^ T cells (49). In addition, the elevated CD25 (high-affinity IL-2R) expression levels, along with their increased IL-2 secretion, speaks for a better overall fitness of CD3^+^CD8^+^CD27^+^CD28^+^ compared to CD3^+^CD8^+^CD27^-^CD28^-^ CART cells.

We show here that CD3^+^CD4^+^ T cells can also be turned into CART killer cells, however, they have a 3- to 6-fold lower killing efficacy when compared to CD3^+^CD8^+^ T cells. Similar to CD8^+^CD27^+^CD28^+^ T cells, CD4^+^CD27^+^CD28^+^ T cells proliferated better and produced more IL-2 and TNF-α when compared to CD4^+^CD27^-^CD28^-^ T cells. However, it is noteworthy that CD4^+^CD27^-^CD28^-^ T cells produced significantly more IFN-γ than CD4^+^CD27^+^CD28^+^ T cells, which may explain their moderately superior killing activity compared with CD4^+^CD27^+^CD28^+^ T cells.

Accordingly, our findings also provide an explanation as to why the lack of CD27 and/or CD28 on T cells has been described to be associated with impaired immuno-surveillance capabilities of non-CART cells, previously (35). While the engagement of CD28 with an agonistic CD28 monoclonal antibody was, in fact “too potent *in vivo*” and induced a highly problematic cytokine storm in six participants of a fist-in-human phase I clinical trial in a previous study (50), engagement of CD27 by varlilumab (CDX-1127), a novel, agonistic, fully human CD27 monoclonal antibody, revealed durable antigen-specific antitumor efficacy (51), by increasing effector T cell numbers with an activated phenotype which was at the expense of naïve and Treg cell numbers in pre-clinical and human phase I and II immunotherapy trials (52). Moreover, conditioning treatment with CD27 mAb in a preclinical model enhanced the expansion and anti-tumor activity of adoptively transferred T cells (53) and by activating T cells recruits and stimulates myeloid cells for enhanced killing of CD27 mAb-opsonized tumors (54). In some CD27 mAb-treated melanoma patients, increased numbers of T cells that recognize melanoma-related antigens were revealed (52). Thus, the expression and active engagement on T cells of CD27 by mAbs has the potential to positively affect adaptive immunotherapy against cancer (55), suggesting conversely that the pathological increase of T cells which lack CD27 expression could be a gradually increasing disadvantage.

Until recently, the best predictors for response to CART cell treatment in r/r DLBCL have been factors not related to immune system function, such as low tumor volume, number of extranodal sites, low serum LDH levels immediately prior to CART cell infusion and a low ECOG performance status (7, 14–16). However, tumor volume/burden lacks specificity because it is a predictor of therapeutic success for the treatment of a large collection of different disease entities and therapies (56). The same holds true for serum LDH levels, which are an established marker of tumor burden, metabolic activity and thus aggressiveness of NHL. Very similar to tumor volume, the serum LDH level has been established as a prognostic factor for the disease course and treatment success of NHL since the 1970s and therefore is also included in the IPI score. Accordingly, while we found elevated serum LDH levels in the overall r/r DLBCL study group (325.9 ± 180.3 U/L), they were lower in OR (236.1 ± 114.0 versus 366.1 ± 162.5; P=0.08) and CR (246.4 ± 132.2 versus 323.9 ± 155.9; P=0.19) patients as compared to non-OR and non-CR patients, respectively, especially when determined at leukapheresis (**Table 1**), although, without reaching statistical significance.

More recently, the search for new biomarkers has turned to studying the nature of the tumor microenvironment, with the intention to identify the mechanistic basis of putative inhibitory factors, followed by the development of strategies for their inhibition/neutralization with, e.g., checkpoint inhibitors (57). These experimental approaches will help us to understand how to pave the way for the facilitated tumor invasion by the infused CART cells and to ultimately steer and support the activation and cytotoxicity of the latter. However, access to the site of tumor cell accumulation in DLBCL for diagnostic purposes, i.e., the bone marrow and/or lymph nodes, demands utterly invasive and thus burdensome procedures (e.g., bone marrow and/or lymph node biopsies). In contrast, the herein described assessment of the levels of peripheral blood CD3^+^CD27^-^CD28^-^ T cells is easy to perform and standardize, also in sequential series of biological samples and thus suitable for daily clinical laboratory routine. In addition, numbers of CD3^+^CD27^-^CD28^-^ T cells can reliably be determined in the leukapheresis product, as well with similar accuracy to peripheral blood (**Figure S6** and **Table S3**).

Which mechanism(s) could be responsible for the down-regulation of CD27 and CD28 on the surface of CD3^+^ T cells in the PB of CART cell non-responders?

The fact that almost 90% of patients expressed elevated levels of HLA-DR^+^ T cells in their circulation (**Table 2**), indicates a possible hyperactivation of the immune system (58), which may be a reflection of the lengthy disease course (35.9 ± 53.8 months) and the associated microbial pressure on the lymphodepleted patients and/or the number of prior therapy lines given (median 3, range 1-11) to our patients. In this study, we found no correlation between the total number of different treatment lines and the number of CD3^+^CD27^-^CD28^-^ T cells in the PB at the time of leukapheresis. However, we found a weak correlation between the number of R-CHOP cycles administered and the number of CD3^+^CD27^-^CD28^-^ T cells (r=0.3931, P=0.0287).

Alternatively, the increased number of HLA-DR^+^ T cells could also be a sign of homeostatic proliferation due to treatment-induced lymphopenia. In this regard, it is important to note that homeostatically proliferating CD8^+^ T cells have been shown to neo-express HLA-DR, but always in conjunction with telomerase activity (36).

One mechanism explaining the loss of CD27 on activated T cells is that these cells tend to upregulate CD70, which is the ligand for CD27 (59). In turn, CD70 up-regulation and interaction with its ligand CD27, either on the same or on adjacent cells, may then lead to reactive downregulation of the latter (60). Similarly, CD28 modulation is known to be the result of cellular activation and replicative senescence (31, 61). Notably, the molecular mechanism(s) leading to CD70 upregulation on T cells during chronic systemic inflammation, such as in lupus erythematosus, are governed by epigenetic changes in T cells, such as histone modifications at the TNFSF7 (CD70) promoter (62) with subsequent downregulation of CD27 on terminally differentiated T effector memory RA cells (TEMRA) (63). CD28^null^ cells were also found to exhibit significant changes in their whole-genome methylation pattern (64) and to receive less signaling through the ERK and JNK pathways, reducing the expression of the DNA methyltransferases Dnmt1 and Dnmt3a, which in turn contributes to the epigenetic downregulation of CD28 expression (65). Taken together, both CD27 and CD28 modulation seem to be governed by several factors, including ligand- and epigenetic/promoter-driven downregulation, all supported by chronic hyperactivation of the immune system.

The fact that low lymphocyte counts are frequently detected in DLBCL patients at initial presentation (66) and that lymphocytopenia after first-line therapy is a predictor of relapse (67) is well known. Therefore, it was not entirely surprising that our patient population suffered from significant lymphopenia. Several reasons can be suggested for the intrinsic activation-induced lymphocyte depletion, such as i) canonical tumor antigen-specific activation by lymphoma cells, ii) cytokine-dependent bystander activation caused by DLBCL-secreted and T cell tropic cytokines like IL-2 and IL-6 (68), or iii) reactivation of latent viruses such as CMV or EBV, which have been shown to be associated with the increased appearance of CD3^+^CD27^-^CD28^-^ PB T cells previously (28). While the first two explanations are the matter of intense research, the latter can be excluded since no CMV and EBV reactivation was observed in our patients.

Previous studies suggested that an increased frequency of CD27^+^CD45RO^-^CD8^+^ T cells at the time of leukapheresis may correlate with sustained remission in patients with chronic lymphocytic leukemia treated with CD19 CART cells (21), in multiple myeloma patients treated with B cell maturation antigen (BCMA)-specific CART cells (22), and very recently in patients with DLBCL (23). The authors suggested that CD27^+^CD45RO^-^CD8^+^ T cells belong to the group of antigen-experienced CD3^+^CD8^+^ T lymphocytes that have long-lasting memory capabilities and improved ability to expand *in vitro* and *in vivo* (21, 22, 24). However, this T cell subset, which according to our algorithm belongs to T cells with a CD3^+^CD27^+^CD28^-^ phenotype (29), was not found to be associated with OR and/or CR in our study (**Table 3**). We considered it important to focus on a combination of T lymphocyte surface markers with proven importance during the T cell activation process, i.e., well-established co-stimulatory molecules, such as CD27 and CD28, rather than the combination of one such marker (CD27) with a purely phenotypic marker, such as CD45RO negativity, which may, in fact, identify more than one T cell phenotype, e.g., naïve T cells and antigen-experienced “stem cell memory” cells (23). *Romero et al.*, showed in healthy individuals that the majority of CD3^+^CD8^+^CD27^-^CD28^-^ T cells is composed of CCR7^-^CD45RA^+^ terminally differentiated T effector memory RA cells (TEMRA), while they clearly also contain a smaller 10-20% fraction of CD27^-^CD28^-^ T cells which belongs to the effector memory (EM) subset. The latter subset is commonly referred to as EM3 cells (37). Indeed, in CART patients at leukapheresis and healthy controls, it turned out that CD3^+^CD8^+^CD27^-^CD28^-^ T cells are also highly enriched for CD45RA^+^CCR7^-^ TEMRA cells (72.3±18.8% in healthy donors vs. 59.2±19.2% in lymphoma patients), the rest of the cells presented with a CD45RA^-^CCR7^-^ EM phenotype, which is compatible with their relationship to EM3 cells (**Figure S4**). Within the CD3^+^CD4^+^ T cell subset, the picture was different. Herein, CD27^-^CD28^-^ T cells are mainly composed of CCR7^-^CD45RA^-^ EM cells belonging to the EM3 phenotype, while the number of TEMRA is usually low to non- existent among CD3^+^CD4^+^ T cells in healthy individuals (**Figure S4**). Thus, when gating on CD3^+^CD27^-^CD28^-^ T cells one reads out the “sum of TEMRA and EM3 T cells” of both CD8^+^ and CD4^+^ T cells, with CD8^+^ T cells mainly contributing to the CD27^-^CD28^-^ phenotype (65.4 ± 23.3% for healthy controls and 59.1 ± 23.8% for patients). A similar picture is seen in typical DLBCL patients (**Figure S4**).

Furthermore, analyzes of activation marker expression on T cells used for *in vitro* experiments confirmed that HLA-DR was clearly expressed on all cell types with a tendency for up-regulation on CD27^-^CD28^-^ T cells as compared to CD27^+^CD28^+^ T cells on CD4 and CD8 T subsets. Moreover,CD69 was upregulated on both CD4^+^ and CD8^+^ CD27^-^CD28^-^ T cells as compared to their CD27^+^CD28^+^ counterparts (**Table S4**). The picture was different for CD25 expression, which was downregulated on CD4^+^CD27^-^CD28^-^ T cells as compared to CD4^+^CD27^+^CD28^+^ T cells. However, no clear sign for the upregulation of exhaustion markers (LAG-3, TIM-3 and PD-1) was evident on *in vitro* tested CART cells (**Table S4**), except TIM-3 on CD8+ T cells.

Comparable albeit slightly different changes were found on cells of patients undergoing leukapheresis. Here, HLA-DR was generally more up-regulated on CD27^-^CD28^-^ T cells when compared to CD27^+^CD28^+^ T cells in patients. CD69 was found to be upregulated more on CD8^+^CD27^-^CD28^-^ T cells as compared to CD8^+^CD27^+^CD28^+^ T cells, while no significant expression of CD69 was found on CD4^+^ T cells (**Figure S9**). CD25 expression was lower in all patients on the CD27^-^CD28^-^ when compared to the CD27^+^CD28^+^ subset. Notably, PD-1 was clearly upregulated on CD4^+^CD27^-^CD28^-^ as compared to CD4^+^CD27^+^CD28^+^ T cells which was in clear contrast to the CD8^+^ subset, in which PD-1 expression was higher on the CD27^+^CD28^+^ T cells when compared to CD27^-^CD28^-^ T cells (**Figure S9**). The latter findings points to a remarkable and potentially functionally relevant dissociation of the expression of co-stimulatory and exhaustion marker molecules in DLBCL patients.

The significant association of low numbers of CD3^+^CD27^-^CD28^-^ T cells in PB at the time of leukapheresis with CR at 3 months with the cut-off of ≤ 18% CD3^+^CD27^-^CD28^-^ T cells to predict CR at 12 months after CART cell treatment seems to be a promising new predictive biomarker. Although our study shows that patients with high numbers of CD3^+^CD27^-^CD28^-^ T cells may not respond as well to CART cell therapy as patients with low numbers of CD3^+^CD27^-^CD28^-^ T cells, we are far from claiming that this circumstance is irreversible. For example, it may well turn out that administration of checkpoint inhibitors at the time of CART cell administration, *e.g.*, against PD-1, could improve the inferior outcome of this group of patients. Of note in that respect, two of our patients with high numbers of differentiated T cells responded to CART cells when pretreated with pembrolizumab (69). Moreover, recent studies have shown that the use of the Bruton’s tyrosine kinase inhibitor ibrutinib (70), or the phosphoinositide-3 kinase inhibitor idelalisib (71, 72) can improve CART cell production in patients with chronic lymphocytic leukemia. Similar effects may be realized in r/r DLBCL in the future.

The better *in vivo* performance of CART cell products containing a low baseline amount of CD3^+^CD27^-^CD28^-^ T cells may also have adverse effects. Patients receiving such T cells may suffer from more treatment-related toxicities after CART cell transfusion because the CART cells may exhibit greater CD19 target cell-dependent proliferation and cytotoxic factor (IFN-γ, TNF-α) production *in vivo* and thus a likely higher killing rate. However, no significant associations were found between the number of CD3^+^CD27^-^CD28^-^ T cells in the leukapheresis product and the occurrence of i) cytokine release syndrome (CRS, r=0.1 and P=0.072), ii) clinical requirements for tocilizumab therapy (r=0.14 and P=0.51), or iii) long-term cytopenias (r=0.16 and P=0.57) (Spearman’s r-tests) in the present study.

Several important limitations of this trial should be considered. During the planning and recruitment phase of this trial no validated flow cytometric assay was available to monitor CART cell expansion *in vivo* and respective binding reagents for reliable monitoring had only become available very recently (73). Therefore, the relationship between the CD3^+^CD27^-^CD28^-^ T cell status determined at leukapheresis and the kinetics of CART cell expansion *in vivo* could not be monitored.

In addition, our study is limited by a small sample size of only 33 patients with 26 patients who received CART cells at least three-month before response assessment. Therefore, larger multi-center studies are certainly needed to confirm our findings in the future. Due to the limited sample size, we were not able to test our biomarker in an independent validation cohort.

It has to be noted that the ethical permission did not include to test CART cells from patients in *in vitro* studies.

Therefore, in the CD19 CART cell *in vitro* studies shown here, T cells of healthy donors were transduced with a CD19-CAR. For that purpose, PBMC from healthy donors were processed for CART cell production using a protocol comparable to that used for the processing of the leukapheresis products from patients, without prior sorting into CD4^+^ and CD8^+^ T cells subsets before transduction and expansion. Accordingly, CD3CD28-bead stimulated PBMC were transduced with the CD19 CAR and further expanded for 14 days. Upon cryopreservation and recultivation, CART cells were further expanded by incubation with irradiated (120 Gy) CD19^+^ TM-LCL cells for 10 days followed by FACS-sorting for CD27 and CD28 expression. TM-LCL cells, while being non-proliferative, are still able to provide the CD19 antigen necessary for antigen-dependent proliferation of CD19 CART cells. They have been successfully used in the past for CD19 CART cell expansion (42). In fact, upon co-culturing with irradiated TM-LCL cells, the authors of this report routinely observed a 18-20-fold expansion of CD19 CART cells within 10 days. Expanded and sorted CART cells were than rested for 7 days followed by determination of their CD27 and CD28 expression status, their antigen-dependent cytotoxicity, proliferative capacity and factor production capabilities. While we did not observe a significant difference in the killing capacity between CD27^+^CD28^+^ and CD27^-^CD28^-^ CART cells, we consider the differences in the proliferative capacity of CD3^+^CD27^+^CD28^+^ CD19 CART cells versus CD3^+^CD27^-^CD28^-^ CD19 CART cells worth reporting, especially since previous studies had already shown that the ability to proliferate and expand well is associated with the expression of T cell clusters harboring upregulated proliferation-associated genes (74). Our study now shows that a similar stratification of T cells can be achieved by virtue of separating T cells according to their surface-expression status of the co-stimulatory molecules CD27 and CD28. It is in line with the linear differentiation model of T cells which has shown previously that CD3^+^CD27^-^CD28^-^ T cells consist of TEMRA and EM3 cells, both belong to the terminally differentiated T effector memory cells with undetectable TREC numbers and short telomers (37). Elevated numbers of this phenotype are not found in healthy individuals (**Figure 2A**), but are a salient feature of individuals with considerable immunological dysregulation (chronic inflammation), such as the one found in r/r DLBCL patients.

In summary, our study has identified that a low number of CD3^+^CD27^-^CD28^-^ T cells is a new biomarker associated with better treatment response to CART cell therapy. This novel insight has the potential to contribute to an improved selection of patients with a high chance of CR after CART cell treatment and/or to form the rational basis for co-medications, such as ibrutinib, at the time of leukapheresis or administration of checkpoint-inhibitors at the time of CART transfusion. Such findings may thus provide the basis for further increasing the success rates of this innovative and potentially curative therapy.

## Data availability statement

The raw data supporting the conclusions of this article will be made available by the authors. Requests to access the datasets should be directed to Dr. Nina Worel at nina.worel@meduniwien.ac.at.

## Ethics statement

The studies involving human participants were reviewed and approved by Ethics Committee of the Medical University of Vienna, EC No.:1422/2015, 1607/2018, 2055/2019, 1290/2020. The patients/participants provided their written informed consent to participate in this study.

## Author contributions

UJ, NW, and WP designed research; NW, KG-P, BK, MS, AT, AR, UK, EP, PS, CS, HH, VG, JH, BS, ML, NS, EF, PW, WR, and IS-K performed research and analyzed data; UJ, GH, PW, NW, and WP supervised experiments and clinical study; WP, NW, KG-P, and UJ wrote the paper. All authors contributed to the article and approved the submitted version.

## Funding

UJ, VG and JH were supported by the Innovative Medicines Initiative (IMI) T2EVOLVE. This project has received funding from the Innovative Medicines Initiative 2 Joint Undertaking under grant agreement No 945393. This Joint Undertaking receives support from the European Union ís Horizon 2020 research and innovation program and EFPIA. The funders had no role in study design, data collection and analysis, decision to publish, or preparation of the manuscript.
