# Supplementary material for: The frequency of differentiated CD3+CD27-CD28- T cells predicts response to CART cell therapy in diffuse large B-cell lymphoma
Source: Front Immunol. 2023 Jan 9;13:1004703. doi: 10.3389/fimmu.2022.1004703 (PMC9868136; doi:10.3389/fimmu.2022.1004703)
Supplement: Supplementary file 1 [file DataSheet_1.docx]

**The frequency of differentiated CD3^+^CD27^-^CD28^-^ T cells predicts response to CART cell therapy in diffuse large B-cell lymphoma**

Nina Worel ^1,^ *, Katharina Grabmeier-Pfistershammer ^2,^*, Bernhard Kratzer ^2,^*, Martina Schlager ^3^, Andreas Tanzmann ^1^, Arno Rottal ^2^, Ulrike Körmöczi ^2^, Edit Porpaczy ^3^, Philipp Staber ^3^, Cathrin Skrabs ^3^, Harald Herkner ^4^, Venugopal Gudiapati ^5^, Johannes Huppa ^5^, Benjamin Salzer ^6^, Manfred Lehner ^6^, Nora Saxenhuber ^3^, Eleonora Friedberg ^3^, Philipp Wohlfarth ^7^, Georg Hopfinger ^7^, Werner Rabitsch ^7^, Ingrid Simonitsch-Klupp ^8^ Ulrich Jäger ^3^, and Winfried F. Pickl ^2^.

^1^ Department of Blood Group Serology and Transfusion Medicine, Medical University of Vienna, Vienna, Austria.

^2^ Institute of Immunology, Center for Pathophysiology, Infectiology and Immunology, Medical University of Vienna, Vienna, Austria.

^3^ Department of Medicine I, Division of Hematology and Hemostaseology, Medical University of Vienna, Vienna, Austria.

^4^ Department of Emergency Medicine, Medical University of Vienna, Vienna, Austria.

^5^ Institute for Hygiene and Applied Immunology, Center for Pathophysiology, Infectiology and Immunology, Medical University of Vienna, Vienna, Austria.

^6^ Christian Doppler Laboratory for Next Generation CAR T Cells, St. Anna Children´s Cancer Research Institute, Vienna, Austria

^7^ Department of Medicine I, Division of Blood and Bone Marrow Transplantation, Medical University of Vienna, Vienna, Austria.

^8^ Department of Pathology, Medical University of Vienna, Vienna, Austria.

* Drs. Worel, Grabmeier-Pfistershammer and Kratzer contributed equally to this article.

Address reprint requests to Dr. Pickl at the Institute of Immunology, Center for Pathophysiology, Infectiology and Immunology, Medical University of Vienna, Lazarettgasse 19, 1090 Vienna, Vienna, Austria, or at winfried.pickl@meduniwien.ac.at

**SUPPLEMENTARY APPENDIX FIGURE LEGENDS**

**Figure S1. Screening, enrollment and disease course of patients receiving CART cell treatment.** Flow diagram shows enrollment strategy and number of patients in respective response groups.

**Figure S2.** **Typical** **distribution of leukocyte populations in a r/r DLBCL patient compared to healthy control subject.** Two representative flow cytometry scatter diagrams showing the distribution of PB cell populations in a typical healthy control subject (HC) and a typical r/r DLBCL patient. The data are representative of 24 healthy control subjects and 31 of 33* r/r DLBCL patients, respectively. The gates were set according to the reactivity of non-binding control monoclonal antibodies. Numbers indicate the percentage of cells in the respective gate or quadrant. *) PB of two patients belonging to the CART responders was not available for analyses at this stage.

**Figure S3. Comparison of the differentiation state of PB CD3^+^ lymphocyte populations in HC, CR and non-CR patients.** Shown are representative two-parameter flow cytometry scatter diagrams analyzing the distribution of PB cell populations with non-differentiated (CD3^+^CD27^+^CD28^+^) versus differentiated (CD3^+^CD27^-^CD28^-^) phenotypes and variants thereof. Representative results of a healthy control subject (HC), a CART cell complete responder (CR) and a CART cell non-complete responder (non-CR, PR, partial response; SD, stable disease; PD, progressive disease) are presented. The gating strategy (FSC/SSC followed by panCD45 gating) based on the reactivity of the non-binding control monoclonal antibodies is shown. The numbers indicate the percentage of cells in the respective gates or quadrants.

**Figure S4.** Analyses of CD27^-^CD28^-^ and CD27^+^CD28^+^ T cells for the expression of CCR7 and CD45RA. **A)** Shown is the gating strategy and the classification of TN, TCM, TEM and TEMRA cells based on the expression of CCR7 and CD45RA. **B)** Shown is the CD27 and CD28 as well as the CCR7 and CD45RA status of a typical healthy control (upper row), complete responder (middle row) and non-complete responder (bottom row). The gates used are indicated on top of each plot. The numbers indicate the percentages of cells in the respective gates or quadrants.

**Figure S5. Quantification of the association between overall response at 3 months and CD3^+^CD27^-^CD28^-^ T cells.** Shown is the unadjusted estimate along with adjusted estimates for clinical and laboratory variables selected by prior clinical importance. The null hypothesis was that there is no association between clinical/laboratory parameters and overall response to CART cell treatment at 3 months after CART infusion. GCB, germinal center B cells; IPI, international prognostic index, NOS, mutations not otherwise specified. **A** shows the association between CART responders vs. CART non-responders and **B** for CR vs non-CR.

**Figure S6. Leukapheresis does not alter the distribution of naïve and differentiated T cell subpopulations.** The graphs compare the distribution of the indicated lymphocyte subpopulations at the time of leukapheresis in PB with that in the leukapheresis product of 31 of 33* individual r/r DLBCL patients (except 26 for CD3^+^CD25^+^ and 28 for CD3^+^HLA-DR^+^) who were scheduled for CART cell therapy. P-values (unpaired t-test) are indicated. *) PB of two patients belonging to the CART cell responders was not available for analyses at this stage.

**Figure S7. Sorting strategy and purity assessment of CD3^+^CD8^+^ CART cells used for *in vitro* assays.** Shown are representative two-parameter flow cytometry scatter plots demonstrating the sorting of the CD8^+^CD27^+^CD28^+^ and CD8^+^CD27^-^CD28^-^ CART cells from the mixed population of expanded CD19^+^ CART cells (upper panel). The transduced CD19^+^ CART cells in the upper row quadrants marked by red and blue rectangles were sorted. The middle panel shows the purity of the sorted CD8^+^CD27^-^CD28^-^ CART cells and the bottom panel shows the purity of sorted CD8^+^CD27^+^CD28^+^ CART cells. Shown is the gating strategy which used FSC/SSC characteristics followed by doublet exclusion with FSC-A/FSC-H leading to positive selection of CD8^+^ CART cells. The percentages of CAR^+^ T cells (gated according to fluorescence minus one control) within the bulk transduced T cells (upper row) and the indicated subsets (middle and lower rows) are shown on the rightmost panels. The numbers indicate the percentages of cells in the respective gates or quadrants.

**Figure S8. Sorting strategy and purity assessment of CD3^+^CD4^+^ CART cells used for *in vitro* assays.** Shown are representative two-parameter flow cytometry scatter plots demonstrating the sorting of the CD4^+^CD27^+^CD28^+^ and CD4^+^CD27^-^CD28^-^ CART cells from the mixed population of expanded CD19^+^ CART cells (upper panel). The transduced CD19^+^ CART cells in the upper row quadrants marked by red and blue rectangles were sorted. The middle panel shows the purity of the sorted CD4^+^CD27^-^CD28^-^ CART cells and the bottom panel shows the purity of sorted CD4^+^CD27^+^CD28^+^ CART cells. Shown is the gating strategy which used FSC/SSC characteristics followed by doublet exclusion with FSC-A/FSC-H leading to positive selection of CD4^+^ CART cells. The percentages of CAR^+^ T cells (gated according to fluorescence minus one control) within the bulk transduced T cells (upper row) and the indicated subsets (middle and lower rows) are shown on the rightmost panels. The numbers indicate the percentages of cells in the respective gates or quadrants.

**Figure S9. Expression of exhaustion markers on T cells of a typical CR and non-CR DLBCL patient at leukapheresis.** Shown are typical two parameter plots for the surface expression of the indicated markers (CD69, CD25, HLA-DR and PD-1) on CD27^+^CD28^+^ and CD27^-^CD28^-^ of CD3^+^CD8^+^ (upper row) and CD3^+^CD4^+^ (lower row) T cells. Gates were set according FMO control stainings.

**Table S1. Time from diagnosis to enrolment, CART cell treatment and further laboratory parameters of patients.** Shown are the mean and median time intervals from diagnosis to leukapheresis, from relapse to CART cell infusion, from relapse to leukapheresis and from leukapheresis to CART cell infusion along with additional potentially relevant laboratory parameters. *) P-values calculated with Student’s t-test, if significant shown in red font; CR, complete remission; PR, partial remission; Hb, hemoglobin; Plt, platelets; LDH, lactate dehydrogenase; CRP, C-reactive protein; B2M, beta-2-microglobulin; GCB, germinal center B cell; ECOG, Eastern cooperative oncology group score; IPI, international performance index; mos=months

**Table S2.** **List of monoclonal antibodies used in CART cell study.** Shown are the specificities, clone names, species of origin, form of conjugation with fluorochrome, source and catalogue number of the respective monoclonal antibodies. Abbreviations: APC, allophycocyanine; FITC, fluorescein isothiocyanate; PerCP, peridinin chlorophyll protein; PE, phycoerythrin.

**Table S3. Leukapheresis does not lead to a significant change of the fractions of CD27^+^CD28^+^ and CD27^-^CD28- lymphocyte subpopulations in the leukapheresis product but increases the percentage of CD3^+^ T cells at the expense of NK-cells.** Shown are relative numbers of the indicated lymphocyte sub-populations within the *) lymphocyte (lines 1-9) or CD3^+^ T cell (lines 10-15) compartment. §) P-values calculated with paired Student’s t-test, if significant shown in red font.

**Table S4**: **Activation and exhaustion maker expression on in vitro differentiated and FACS sorted CART cells.**

Shown are relative numbers of the indicated lymphocyte sub-populations as mean ± standard deviation.

**Figure S1**

33 patients were enrolled in the study

26 patients received a CART cell infusion

and were further analyzed

5 patients deceased

2 patients received

other treatment

15 patients had a complete

(11) or partial (4) remission

11 patients had stable

disease, disease

progression or deceased

**Figure S2**

**Figure S3**

**Figure S4**

**Figure S5**

**A**

**B**

**Figure S6**

**Figure S7**

**
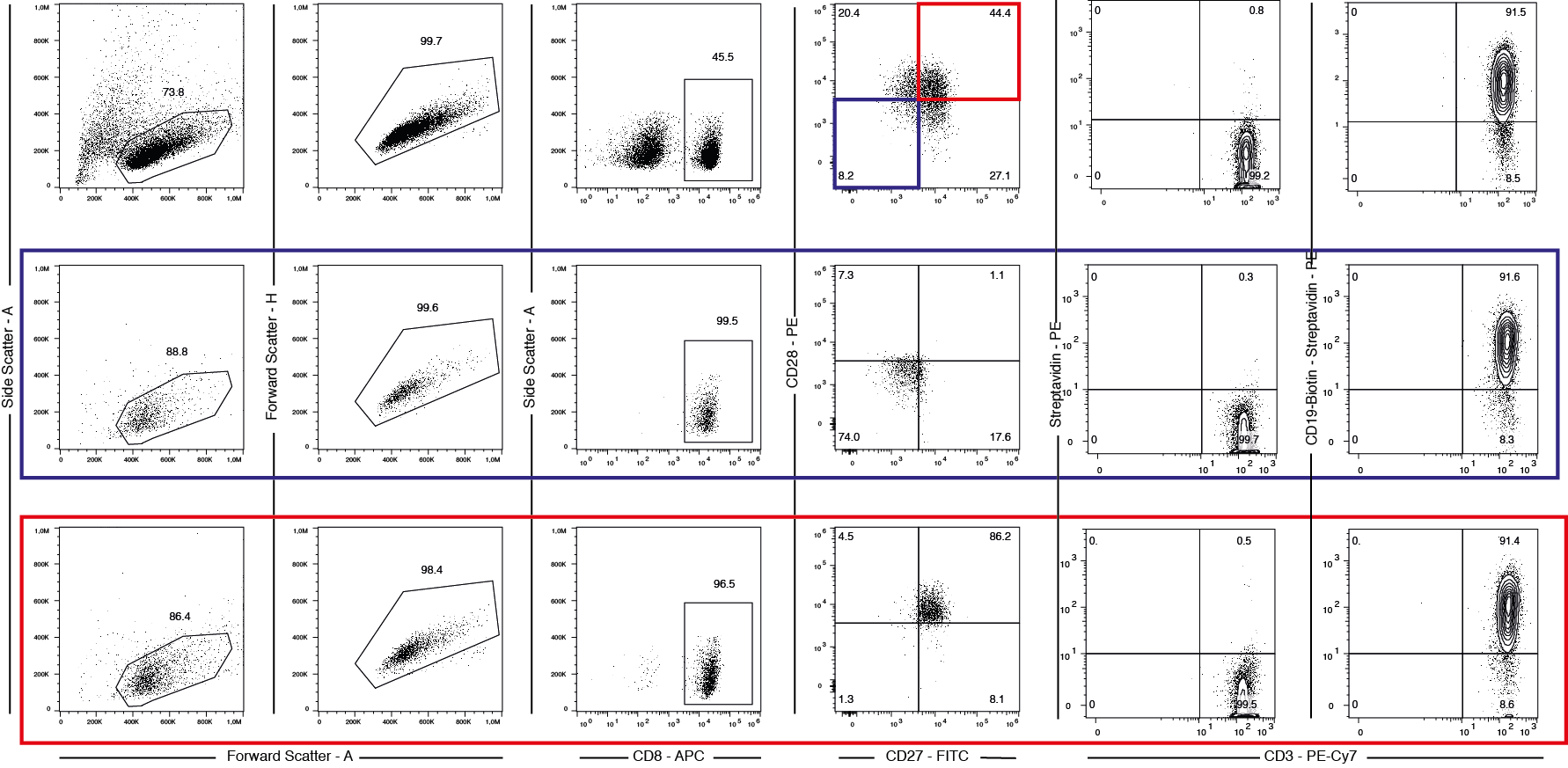
**

**Figure S8**
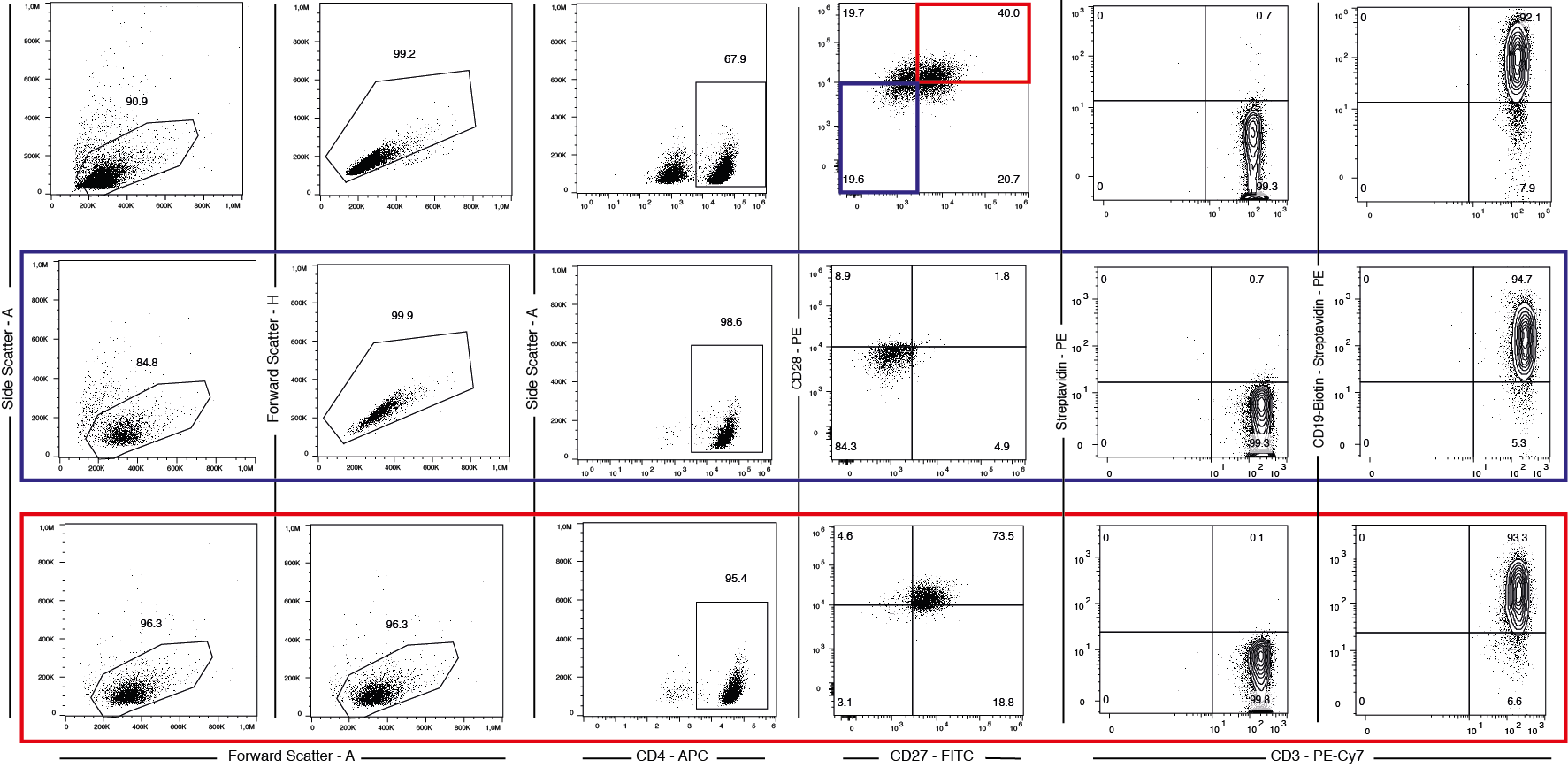


**Figure S9**

**
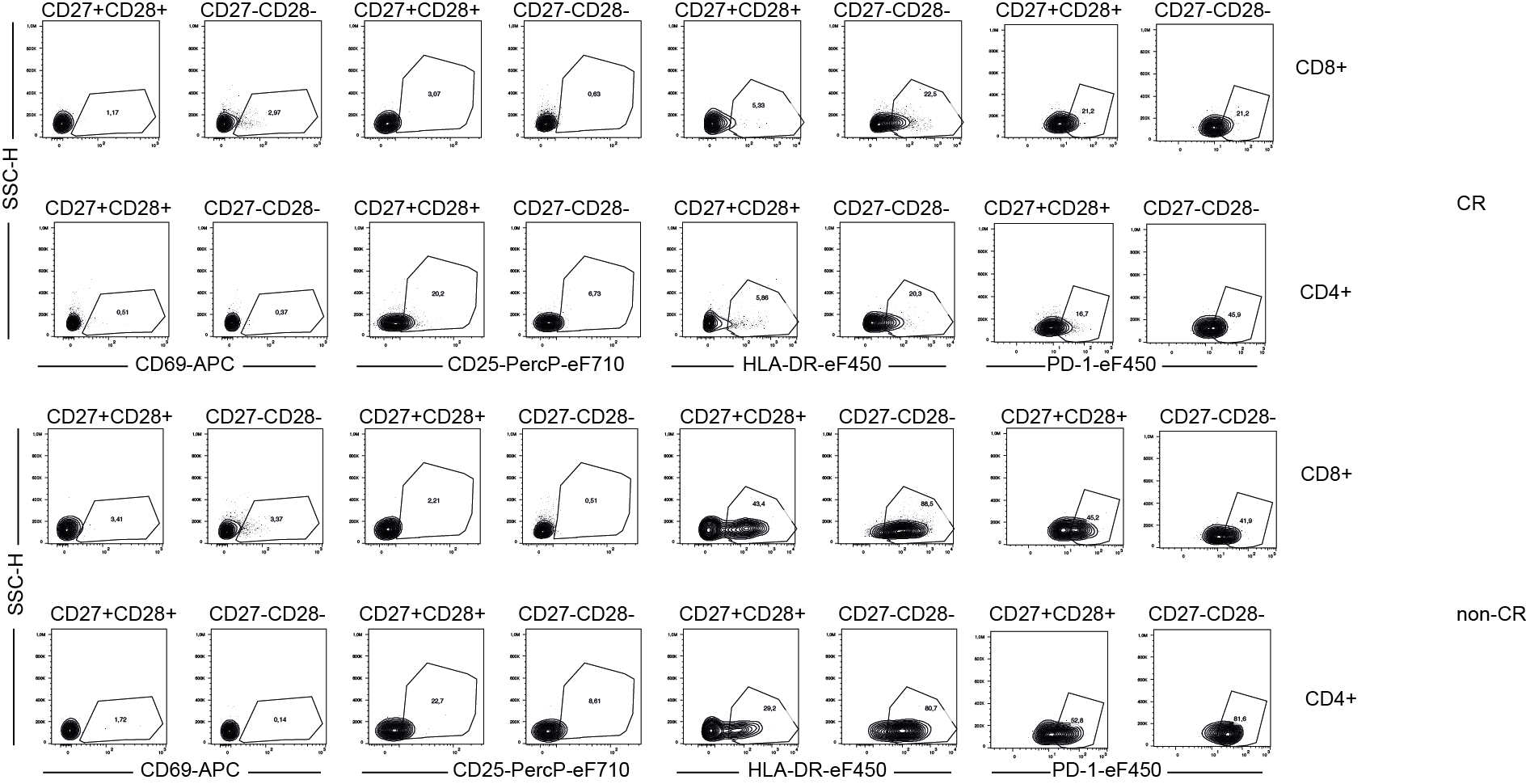
**

**Table S1. Time from diagnosis to enrolment, CART cell treatment and further laboratory parameters of patients.**

|  | **All DLBCL patients enrolled in study** | **Patients who received CART cell treatment (n=26; 78.8%)** | | | | | |
| --- | --- | --- | --- | --- | --- | --- | --- |
|  |  | **3 mos responders**  **(CR+PR)** | **3 mos non-responders** | **P-value^*^** | **3 mos CR** | **3 mos Non-CR** | **P-value** |
| **No. of patients (%)** | **33 (100)** | **15 (45.5)** | **11 (33.3)** |  | **11 (33.3)** | **15 (45.5)** |  |
| *Duration diagnosis of DLBCL to leukapheresis* |  |  |  |  |  |  |  |
| Mean±SD in months | 29.4±49.6 | 49.0±70.5 | 16.7±13.6 | 0.15 | 57.7±81.2 | 19.5±18.7 | 0.09 |
| Median in months (range) | 18.0 (3.7-266.4) | 24.7 (5.5-266.4) | 10.9 (7.0-53.6) |  | 24.7 (6.5-266.4) | 10.9 (5.5-69.5) |  |
| *Duration relapse to CART* |  |  |  |  |  |  |  |
| Mean±SD in months | 4.9±2.2 | 4.9±2.3 | 5.0±2.2 | 0.85 | 5.2±2.6 | 4.8±2.0 | 0.65 |
| Median in months (range) | 4.7 (1.9-11.5) | 4.9 (1.9±11.5) | 4.5 (2.2-8.9) |  | 5.0 (1.9-11.5) | 4.1 (2.2-8.9) |  |
| *Duration relapse to leukapheresis* |  |  |  |  |  |  |  |
| Mean±SD in months | 2.4±1.8 | 1.7±1.2 | 2.6±2.0 | 0.19 | 2.0±1.3 | 2.2±1.9 | 0.80 |
| Median in months (range) | 1.8 (0.3-7.2) | 1.3 (0.4-4.0) | 1.8 (0.3-7.2) |  | 1.5 (0.6-4.0) | 1.7 (0.3-7.2) |  |
| *Duration leukapheresis to CART* |  |  |  |  |  |  |  |
| Mean±SD in months | 3.3±2.9 | 3.9±3.6 | 2.6±0.9 | 0.25 | 4.2±4.2 | 2.7±1.1 | 0.18 |
| Median in months (range) | 2.7 (1.2-14.1) | 2.8 (1.2-14.1) | 2.6 (1.3-4.1) |  | 2.8 (1.2-14.1) | 2.6 (1.3-5.1) |  |
| *Duration diagnosis of DLBCL to CART cells* |  |  |  |  |  |  |  |
| Mean±SD in months | 35.9±53.8 | 48.4±68.1 | 18.9±13.8 | 0.17 | 55.1±77.7 | 21.8 ±18.6 | 0.12 |
| Median in months (range) | 20.0 (4.6-266.5) | 27.4 (4.6-266.5) | 13.3 (9.0-56.9) |  | 27.4 (4.6-266.5) | 13.3 (8.0-71.7) |  |
| **Laboratory parameters** |  |  |  |  |  |  |  |
| *At leukapheresis* |  |  |  |  |  |  |  |
| Hb (12-18 g/dL), mean±SD | 11.4±1.6 | 11.7±1.3 | 11.8±1.6 | 0.96 | 11.5±1.4 | 11.9±1.4 | 0.43 |
| Plt (150-350 x 10^9^/L), mean±SD | 220.0±116.4 | 182.7±74.7 | 251.3±165.1 | 0.17 | 175.0±49.3 | 238.6±153.7 | 0.20 |
| Albumin (35-50 g/L), mean±SD | 41.7±3.3 | 43.0±2.4 | 41.9±3.2 | 0.33 | 43.3±2.7 | 42.1±2.8 | 0.29 |
| IgG (n=29) (650-1700 mg/dL), Mean±SD | 636.6±206.5 | 609.0±216.3 | 653.1±187.1 | 0.63 | 626.5±221.2 | 627.5±193.4 | 0.99 |
| IgM (n=29) (40-230 mg/dL), mean±SD | 49.3±69.1 | 68.2±100.0 | 40.2±21.2 | 0.42 | 77.8±112.7 | 39.2±22.4 | 0.26 |
| IgA (n=29) (70-400 mg/dL), mean±SD | 127.5±82.5 | 108.2±66.6 | 120.6±89.8 | 0.71 | 103.6±66.0 | 121.4±83.0 | 0.59 |
| *At CART cell infusion* |  |  |  |  |  |  |  |
| Hb (n=26) (12-18 g/dL), Mean±SD | 10.6±1.8 | 11.0±1.6 | 10.1±1.9 | 0.23 | 10.6±1.6 | 10.6±2.0 | 0.98 |
| Plt (n=26) (150-350 x 10^9^/L), mean±SD | 160.9±102.7 | 163.1±85.4 | 157.9±126.9 | 0.90 | 158.9±71.0 | 162.4±123.3 | 0.93 |
| Albumin (n=26) (35-50 g/L), mean±SD | 36.8±5.3 | 39.4±3.6 | 33.5±5.4 | 0.03 | 39.5±3.1 | 35.1±5.8 | 0.37 |
| IgG (n=23) (650-1700 mg/dL), mean±SD | 501.3±204.0 | 560.6±189.1 | 409.1±191.3 | 0.08 | 609.4±120.1 | 418.2±213.5 | 0.02 |
| IgM (n=23) (40-230 mg/dL), mean±SD | 41.9±60.3 | 53.7±75.5 | 23.5±11.8 | 0.25 | 63.9±87.6 | 25.0±14.3 | 0.13 |
| IgA (n=23) (70-400 mg/dL), mean±SD | 77.1±65.4 | 69.6±62.2 | 88.9±72.2 | 0.50 | 69.2±64.5 | 83.2±67.9 | 0.62 |

Shown are the mean and median time intervals from diagnosis to leukapheresis, from relapse to CART cell infusion, from relapse to leukapheresis and from leukapheresis to CART cell infusion along with additional potentially relevant laboratory parameters. *) P-values calculated with Student’s t-test, if significant shown in red font; CR, complete remission; PR, partial remission; Hb, hemoglobin; Plt, platelets; LDH, lactate dehydrogenase; CRP, C-reactive protein; B2M, beta-2-microglobulin; GCB, germinal center B cell; ECOG, Eastern cooperative oncology group score; IPI, international performance index; mos=months

**Table S2. List of monoclonal antibodies used in CART cell study**

| **Specificity** | **Clone Name** | **Species** | **conjugate** | **Source** | **Catalogue Number** | |
| --- | --- | --- | --- | --- | --- | --- |
| IgG2a | 4H1-A7 | mouse | FITC | Nordic Mubio | | GCT202 |
| IgG1 | VI-AP | mouse | PE | Nordic Mubio | | GCT202 |
| IgG1 | MOPC-21 | mouse | APC | Biolegend | | 400120 |
| IgG1 | MOPC-21 | mouse | PerCP | Biolegend | | 400148 |
| CD3 | UCHT1 | mouse | PerCP | Biolegend | | 300428 |
| CD3 | UCHT1 | mouse | APC | BD Pharmingen | | 561811 |
| CD4 | RPA-T4 | mouse | FITC | Biolegend | | 300506 |
| CD8 | RPA-T8 | mouse | PE | BD Pharmingen | | 555367 |
| CD14 | MEM18 | mouse | PE | Nordic Mubio | | GM-4093 |
| CD16 | 3G8 | mouse | PE | Life Technologies | | MHCD1604 |
| CD19 | SJ25-C1 | mouse | FITC | Life Technologies | | MHCD19014 |
| CD25 | CD25-3G10 | mouse | PE | Invitrogen | | MHCD2504 |
| CD27 | O323 | mouse | FITC | Biolegend | | 302806 |
| CD28 | CD28.2 | mouse | PE | Biolegend | | 302908 |
| CD45pan | HI30 | mouse | APC | Invitrogen | | MHCD4505 |
| CD56 | NCAM16.2 | mouse | PE | BD | | 345812 |
| HLA-DR | L243 | mouse | FITC | Biolegend | | 307604 |
| TCRa/b | IP26 | mouse | PE | ExBio | | 1P-607-T100 |
| TCRg/d | B1 | mouse | FITC | BD Pharmingen | | 559878 |
| CD3 | UCHT1 | mouse | PE-Cy7 | Thermo Fisher Scientific | | 25-00038-42 |
| CD4 | RPA-T4 | mouse | PE-eFluor610 | Thermo Fisher Scientific | | 61-0049-42 |
| CD8 | RPA-T8 | mouse | AF700 | Thermo Fisher Scientific | | 56-0088-42 |
| CD8 | RPA-T8 | mouse | eFluor450 | Thermo Fisher Scientific | | 48-0088-42 |
| CD45RA | HI100 | mouse | AF700 | Biolegend | | 304120 |
| CCR7 | 3D12 | mouse | APC | Thermo Fisher Scientific | | 17-1979-42 |
| CD25 | CD25-4E3 | mouse | PerCP-eFluor710 | Thermo Fisher Scientific | | 46-0257-42 |
| CD28 | CD28.2 | mouse | APC-eFluor780 | Thermo Fisher Scientific | | 47-0289-42 |
| CD69 | FN50 | mouse | APC | Biolegend | | 310910 |
| LAG-3 | 3DS223H | mouse | PE | Thermo Fisher Scientific | | 12-2239-42 |
| TIM-3 | 344823 | rat | APC | R&D | | FAB2365A |
| PD-1 | EH12.2H7 | mouse | BV421 | Biolegend | | 329920 |
| HLA-DR | L243 | mouse | eF450 | Thermo Fisher Scientific | | 48-9952-42 |
| CD19 CAR Detection Reagent |  |  | Biotin | Miltenyi | | 130-129-550 |

Shown are the specificities, clone names, species of origin, form of conjugation with fluorochrome, source and catalogue number of the respective monoclonal antibodies. Abbreviations: APC, allophycocyanine; FITC, fluorescein isothiocyanate; PerCP, peridinin chlorophyll protein; PE, phycoerythrin ,AF, Alexa Fluor, BV, brilliant violet.

**Table S3. Leukapheresis does not lead to a significant change of the fractions of CD27^+^CD28^+^ and CD27^-^CD28^-^ lymphocyte subpopulations in the leukapheresis product but increases the percentage of CD3^+^ T cells at the expense of NK-cells.**

| **Percent of lymphocytes** | **PB of r/r DLBCL**  **patients**  **(n=31; 94.0%)** | **Leukapheresis product of r/r DLBCL patients**  **(n=31; 94.0%)** | **P-value**^§^ |
| --- | --- | --- | --- |
| CD3^+^ | 76.0±14.4**^*^** | 79.5±13.5 | 0.002 |
| CD3^+^CD4^+^ | 32.3±10.3 | 31.5±12.2 | 0.40 |
| CD3^+^CD8^+^ | 40.5±14.9 | 44.5±16.7 | <0.001 |
| CD3^+^CD25^+^ | 17.2±10.7 | 18.0.±11.1 | 0.41 |
| CD3^+^HLA-DR^+^ | 28.9±16.8 | 24.3±16.6 | 0.001 |
| CD3^+^TCRab^+^ | 67.9±14.2 | 71.0±14.2 | 0.01 |
| CD3^+^TCRgd^+^ | 6.0±5.3 | 8.1±6.3 | 0.03 |
| CD19^+^ | 1.1±2.5 | 0.7±1.8 | 0.04 |
| CD3^-^CD56^+^CD16^+^ | 17.9±12.1 | 15.7±13.0 | 0.007 |
| CD3^+^CD27^+^CD28^+^ | 48.6±18.4 | 49.1±17.7 | 0.66 |
| CD3^+^CD27^-^ | 38.6±19.2 | 37.9±18.7 | 0.44 |
| CD3^+^CD28^-^ | 41.7±19.6 | 40.1±19.2 | 0.15 |
| CD3^+^CD27^+^CD28^-^ | 13.0±8.5 | 13.0±8.8 | 0.97 |
| CD3^+^CD27^-^CD28^+^ | 9.9±7.3 | 10.8±8.2 | 0.001 |
| CD3^+^CD27^-^CD28^-^ | 28.7±19.0 | 27.1±18.7 | 0.07 |

Shown are relative numbers of the indicated lymphocyte sub-populations within the *) lymphocyte (lines 1-9) or CD3^+^ T cell (lines 10-15) compartment. §) P-values calculated with paired Student’s t-test, if significant shown in red font.

**Table S4. Activation and exhaustion maker expression on in vitro differentiated and FACS sorted CAR T cells**

| **Activation marker** | **% of CD8^+^CD27^-^CD28^-^ cells** | **% of CD8^+^CD27^+^CD28^+^ cells** | **% of CD4^+^CD27^-^CD28^-^ cells** | **% of CD4^+^CD27^+^CD28^+^ cells** |
| --- | --- | --- | --- | --- |
| HLA-DR | 28.1±24.3 | 17.5±11.8 | 30.4±15.9 | 19.7±6.2 |
| CD69 | 16.8±25.8 | 10.7±11.7 | 16.4±21.4 | 11.2±15.4 |
| CD25 | 0.6±0.5 | 0.8±1.0 | 1.1±0.1 | 1.3±1.6 |
| LAG-3 | 0.2±0.3 | 0.1±0.2 | 0.6±0.8 | 0.4±0.3 |
| TIM-3 | 0.7±0.4 | 0.9±0.1 | 0.5±0.3 | 0.9±0.0 |
| PD-1 | 1.4±1.2 | 1.1±1.0 | 5.0±1.9 | 5.3±1.5 |

Shown are relative numbers of the indicated lymphocyte sub-populations as mean ± standard deviation.
